# Diversity in spatial scope of contrast adaptation among mouse retinal ganglion cells

**DOI:** 10.1152/jn.00529.2017

**Published:** 2017-09-13

**Authors:** Mohammad Hossein Khani, Tim Gollisch

**Affiliations:** ^1^University Medical Center Göttingen, Dept. of Ophthalmology and Bernstein Center for Computational Neuroscience Göttingen, Göttingen, Germany; and; ^2^International Max Planck Research School for Neuroscience, Göttingen, Germany

**Keywords:** retina, mouse, ganglion cell, receptive field, contrast adaptation

## Abstract

Retinal ganglion cells adapt to changes in visual contrast by adjusting their response kinetics and sensitivity. While much work has focused on the time scales of these adaptation processes, less is known about the spatial scale of contrast adaptation. For example, do small, localized contrast changes affect a cell’s signal processing across its entire receptive field? Previous investigations have provided conflicting evidence, suggesting that contrast adaptation occurs either locally within subregions of a ganglion cell’s receptive field or globally over the receptive field in its entirety. Here, we investigated the spatial extent of contrast adaptation in ganglion cells of the isolated mouse retina through multielectrode-array recordings. We applied visual stimuli so that ganglion cell receptive fields contained regions where the average contrast level changed periodically as well as regions with constant average contrast level. This allowed us to analyze temporal stimulus integration and sensitivity separately for stimulus regions with and without contrast changes. We found that the spatial scope of contrast adaptation depends strongly on cell identity, with some ganglion cells displaying clear local adaptation, whereas others, in particular large transient ganglion cells, adapted globally to contrast changes. Thus, the spatial scope of contrast adaptation in mouse retinal ganglion cells appears to be cell-type specific. This could reflect differences in mechanisms of contrast adaptation and may contribute to the functional diversity of different ganglion cell types.

**NEW & NOTEWORTHY** Understanding whether adaptation of a neuron in a sensory system can occur locally inside the receptive field or whether it always globally affects the entire receptive field is important for understanding how the neuron processes complex sensory stimuli. For mouse retinal ganglion cells, we here show that both local and global contrast adaptation exist and that this diversity in spatial scope can contribute to the functional diversity of retinal ganglion cell types.

## Abstract (precis)

Understanding whether adaptation of a neuron in a sensory system can occur locally inside the receptive field or whether it always globally affects the entire receptive field is important for understanding how the neuron processes complex sensory stimuli. For mouse retinal ganglion cells, we here show that both local and global contrast adaptation exist and that this diversity in spatial scope can contribute to the functional diversity of retinal ganglion cell types.

neurons throughout different sensory systems need to operate under a wide variety of stimulus conditions, and they therefore often adapt to mean, variance, and other statistics of the encountered distribution of stimulus intensities. Variance adaptation in particular has received much attention in different sensory systems, including the auditory (Dean et al. 2005; Kvale and Schreiner 2004; Nagel and Doupe 2006), somatosensory (Garcia-Lazaro et al. 2007; Maravall et al. 2007), and visual system (Carandini and Ferster 1997; Dhruv and Carandini 2014; Levy et al. 2013; Maffei et al. 1973; Movshon and Lennie 1979; Shapley and Victor 1978). In the visual system, variance adaptation manifests itself as adaptation to visual contrast, which starts in the retina (Baccus and Meister 2002; Chander and Chichilnisky 2001; Kim and Rieke 2001; Liu and Gollisch 2015; Rieke 2001; Shapley and Victor 1978; Smirnakis et al. 1997) and is passed on to higher visual areas (Solomon et al. 2004). After an increase in contrast, retinal ganglion cells exhibit reduced sensitivity as well as altered characteristics of temporal stimulus filtering, becoming more band-pass-like with faster responses.

Several previous studies have investigated the temporal dynamics of these adaptation phenomena, showing that changes in temporal filtering are nearly instantaneous (Baccus and Meister 2002; Shapley and Victor 1978), whereas sensitivity changes occur on multiple time scales (Baccus and Meister 2002; Demb 2002, 2008; Smirnakis et al. 1997), which themselves contain stimulus-dependent dynamics (Wark et al. 2009). Much less, however, is known about the spatial scope of contrast adaptation, that is, whether local changes in contrast affect signal processing only in those parts of a ganglion cell’s receptive field where contrast actually changes or whether the adaptation effects spread globally across the receptive field. Understanding whether contrast adaptation occurs locally or globally in the receptive field is important for capturing how ganglion cells encode stimuli with complex spatial structure, such as natural stimuli (Rieke and Rudd 2009). For example, a high-contrast object covering part of a ganglion cell’s receptive field will induce adaptation. Whether this adaptation affects the processing of a second object at a different location inside the receptive field (or of the same object after it has moved to a different location) depends on whether adaptation acts on a local or global scope. It is worth noting in this regard that ganglion cell receptive fields in mouse retina typically span several degrees of visual angle (Koehler et al. 2011), providing ample space for spatial structure and multiple objects inside a single receptive field. Thus, knowing the spatial scope of adaptation is important for understanding how a neuron integrates sensory information across its receptive field and for building models that capture responses to complex stimuli (Real et al. 2017; Rieke and Rudd 2009).

Mechanistically, both local synaptic processes (Jarsky et al. 2011; Kim and Rieke 2001; Manookin and Demb 2006; Ozuysal and Baccus 2012) and global ganglion cell-inherent effects (Kim and Rieke 2001, 2003; Weick and Demb 2011) have been implicated in retinal contrast adaptation. From a functional perspective, different studies have provided indirect evidence for either local or global contrast adaptation. The independence of contrast adaptation on the spatial phase of a stimulating grating (Shapley and Victor 1978) and the similarity to suppressive effects of peripheral stimuli (Shapley and Victor 1979, 1981) observed in cat retina indicated a global scope of contrast adaptation. On the other hand, transient response peaks measured in rabbit retina after switching the location of a small stimulation patch was interpreted as a consequence of local adaptation (Brown and Masland 2001). For the salamander retina, we recently showed that global contrast adaptation effects predominate (Garvert and Gollisch 2013). Here, we extend this analysis to the mammalian retina, by recording spikes with multielectrode arrays from isolated mouse retina under stimulation that contains local changes in contrast.

## METHODS

### 

#### Tissue preparation and electrophysiology.

We used retinas of adult mice (C57BL/6; aged 7–8 wk) of either sex. All procedures conformed to national and institutional guidelines and were approved by the institutional animal care committee of the University Medical Center Göttingen (protocol number T11/35). After dark adaptation for ~1 h, animals were euthanized by cervical dislocation, and both eyes were removed quickly. The eyes were dissected under infrared illumination at a stereomicroscope equipped with infrared goggles. Cornea, lens, and vitreous humor were carefully removed before dissecting each eyecup into two halves. One retina half was then isolated from the pigment epithelium and placed onto a multielectrode array (MultiChannel Systems, Reutlingen, Germany; 252 or 60 channels, 30 or 10 µm electrode diameter, and 100 µm minimum electrode spacing) with ganglion cells facing the electrodes. The other retina pieces were stored for later recordings in oxygenated (95% O_2_-5% CO_2_) Ames medium (Sigma-Aldrich, Munich, Germany), supplemented with 22 mM NaHCO_3_ and 6 mM d-glucose.

During recording, the retina was perfused with oxygenated Ames medium, heated to a constant temperature of around 32–33°C with an inline heater (PH01, MultiChannel Systems, Reutlingen, Germany) and a heating plate below the multielectrode array. The signals were amplified, band-pass filtered between 300 Hz and 5 kHz, and stored digitally at 10 kHz (252-electrode arrays) or 25 kHz (60-electrode arrays). We extracted spike trains of individual ganglion cells with a custom-made spike-sorting program, based on a Gaussian mixture model and an expectation-maximization algorithm (Pouzat et al. 2002). For further analysis, we only included well-separated units with clear refractory period. In total, 398 ganglion cells from 17 retinas were used in the final analysis.

#### Visual stimulation.

Visual stimuli were generated and controlled through custom-made software, written in C++ and using the OpenGL library. Stimuli were displayed at 60 Hz refresh rate on a gamma-corrected white OLED display (800 × 600 pixels; eMagin, Bellevue, WA), which was demagnified to 7.5 µm×7.5 µm per pixel and projected onto the photoreceptor layer of the retina through a telecentric lens (Edmund Optics, Karlsruhe, Germany). All stimuli were presented on a gray background in the photopic range with rod isomerization rates between 1.1×10^4^ and 1.4×10^4^ R*·rod^−1^·s^−1^ (with variations stemming from the use of different experimental setups).

#### Receptive field measurements.

We measured the receptive field for each ganglion cell by calculating the spike-triggered average in response to a spatiotemporal binary white-noise stimulus (100% contrast) on a checkerboard layout with subfields of 60 µm × 60 µm, updated at 30 Hz. The spike-triggered average was separated into its spatial and temporal components by singular-value decomposition (Gauthier et al. 2009; Wolfe and Palmer 1998). The spatial component was then fitted by a two-dimensional Gaussian function, and the receptive-field diameter was defined as the diameter of a circle with equal area as inside the 1.5-σ contour of the Gaussian fit. The temporal component was used to classify the cells as either ON cells or OFF cells, depending on the polarity of the strongest peak in the temporal component.

#### Spatial contrast adaptation stimulus.

**Fig. 1.**
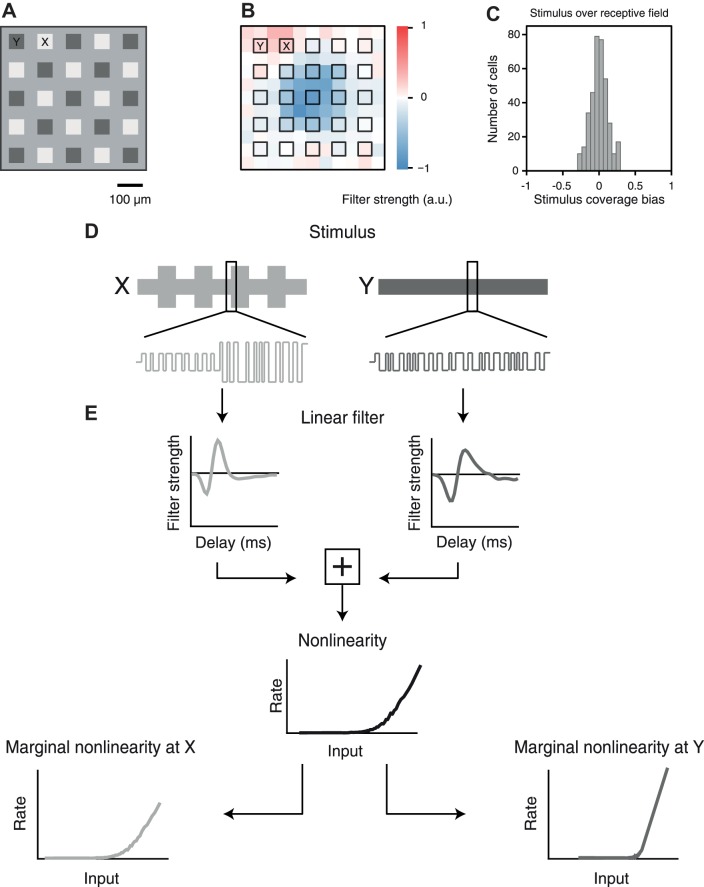
Schematic depiction of visual stimulus and analysis for assessing the spatial scope of contrast adaptation. *A*: stimulus frame with alternating regions *X* (here bright squares) and *Y* (here dark squares) on gray background. *B*: receptive field of a sample mouse ganglion cell, in relation to the stimulus layout. *C*: distribution of stimulus coverage bias for all recorded cells. The stimulus coverage bias measures the relative contributions of components *X* and *Y* in covering the receptive field center of a cell. *D*: schematic representation of the stimulus sequences at *X* and *Y*, showing the envelope of light intensities, indicating contrast changes at *X* every 40 s (*top*), and sample traces of the binary white-noise sequences (*bottom*, not to scale). *E*: linear-nonlinear model for assessing temporal filtering and sensitivity at *X* and *Y*. The linear filtering stage consists of 2 temporal filters, one for *X* and one for *Y*, whose outputs are summed. The nonlinear stage is further analyzed by computing marginal nonlinearities for *X* and *Y*, which represent the effect of one set of locations on the firing rate, averaged over the contributions from the other location.

To assess the spatial scope of contrast adaptation within the receptive field of each recorded ganglion cell, we used a stimulus layout with two sets of alternatingly arranged square-shaped spatial subfields, denoted as *locations X* and *Y* (Fig. 1*A*), similar to our previous investigations in salamander retina (Garvert and Gollisch 2013). Subfields were 60 µm wide and separated by corridors of the same width at the background light level. The subfield size was chosen so that each ganglion cell receptive field typically spanned multiple stimulus subfields (Fig. 1*B*).

We checked that both sets of locations, *X* and *Y*, typically covered approximately equal areas of a receptive field in the following way: We computed for each cell the effective stimulus areas *A_X_* and *A_Y_* by weighting each pixel of the stimulus screen according to the Gaussian fit of the cell’s receptive field and then summed within the 1.5-σ contour all those pixel values that contributed to *locations X* and *Y*, respectively. We then calculated a stimulus coverage bias index as *I*_coverage bias_=(*A_X_*−*A_Y_*)/(*A_X_*+*A_Y_*), which is zero if *X* and *Y* contributed equal area and approaches +1 or −1 if the receptive field coverage was dominated by *X* or *Y*, respectively. The distribution of *I*_coverage bias_ for all recorded ganglion cells shows that the values were narrowly distributed around zero (Fig. 1*C*), indicating that each individual ganglion cell was about equally susceptible to stimulation at *X* or *Y*. Bipolar cells, on the other hand, which provide the excitatory input to the ganglion cells, have smaller receptive fields (Berntson and Taylor 2000; Schwartz et al. 2012), comparable to or smaller than the stimulus subfields chosen here, so that an individual bipolar cell was typically stimulated by only one of the subfields.

Two independent white-noise sequences of light intensities were used as stimuli at *locations X* and *Y* so that stimuli were identical at locations from the same set, but independent across the two sets. For each set of locations, the white-noise sequence was drawn from a binary distribution with values *I*_high_ and *I*_low_ at 60 Hz. The mean light intensity (*I*_high_+*I*_low_)/2 was always set equal to the background level. The contrast level, defined as *C*=(*I*_high_−*I*_low_)/(*I*_high_+*I*_low_), was set to either a high value, *C*=100%, or a low value, *C*=20%. For the primary stimulus applied in this work, contrast levels were switched every 40 s between a high-low condition, with high contrast at *locations X* and low contrast at *locations Y*, and a low-low condition, with low contrast at both sets of locations (Fig. 1*D*). For comparison, we also analyzed responses to stimuli that alternated the high contrast between *locations X* and *Y* by switching between the high-low condition and a low-high condition that had low contrast at *locations X* and high contrast at *locations Y*.

To compare the activity level of each ganglion cell during the low-low and the high-low contrast condition, we measured the average firing rates *R*_low-low_ and *R*_high-low_ during the last 30 s of the corresponding condition and computed the normalized change in steady-state firing rate for each cell as Δ*R*_steady-state_ = (*R*_high-low_−*R*_low-low_)/(*R*_high-low_+*R*_low-low_).

#### Analysis of temporal filtering.

To assess the effects of contrast adaptation, we analyzed response properties of retinal ganglion cells in the framework of the linear-nonlinear (LN) model (Berry and Meister 1998; Chichilnisky 2001; Kim and Rieke 2001; Korenberg and Hunter 1986; Sakai et al. 1988). This model consists of two stages, a linear stimulus filter and a static nonlinearity, which converts the filter output into a firing rate (Fig. 1*E*). In our case, the stimulus filter can be thought of as a spatiotemporal filter with two spatial components, corresponding to the sets of *locations X* and *Y*, each with its own temporal filter shape. For convenience, we will refer to these temporal filter shapes at the two spatial components simply as the filters at *locations X* and *Y*, respectively. These filters describe how the ganglion cell integrates each of the two stimulus components over time. For each contrast condition, the filters were obtained as the spike-triggered average (STA) over a window of 670 ms before each spike. To do so, the stimulus was temporally upsampled by a factor of 17 to a resolution of ~1 ms, and spike trains were binned at the same temporal resolution. Note that the application of the STA approach generally requires a spherically symmetric stimulus distribution (Chichilnisky 2001; Paninski 2003; Schwartz et al. 2006), such as a Gaussian distribution. Yet, even though the applied binary stimulus distribution is not spherically symmetric, the use of the STA still works in practice because the obtained filters show contributions over much longer periods than the stimulus sampling. Thus, the high dimensionality of contributing stimulus components makes the stimulus distribution effectively symmetric around the relevant filter (Chichilnisky 2001).

For comparison of filter shapes, we normalized each filter to unit Euclidean norm, so that the sum of squares equaled unity. To measure the shift in time-to-peak, we fitted the 50-ms region around the maximum value (for ON cells) or minimum value (for OFF cells) with a second-order polynomial, from which the peak time was calculated. The shift in time-to-peak was defined as Δ*P* =(*P*_low-low_−*P*_high-low_). The temporal filters typically had a biphasic shape, with the first peak followed by a second peak of opposite polarity. To analyze effects of contrast changes on the filter shape, we therefore calculated for each filter a biphasic index (Garvert and Gollisch 2013; Zaghloul et al. 2007) as the absolute value of the ratio of the size of the second vs. first filter peak. The filter peak sizes were again determined from second-order polynomial fits, analogous to the calculation of the time-to-peak.

#### Assessing filter changes.

To measure how strongly filter shapes changed between different contrast conditions, we computed a filter dissimilarity index, *FDI*, for two filters *F*_1_ and *F*_2_ obtained from the same cell under different contrast conditions. To do so, we treated the filters as vectors, calculated their scalar product, and subtracted the value from unity, $FDI=1-{\overset{⃗}{F}}_{1}\cdot {\overset{⃗}{F}}_{2}$. To reduce the influence of noise in the filter estimates on this measure, we restricted the filters for this analysis to the range from 50 to 250 ms and normalized the restricted filters again to unit Euclidean norm. This range captured most of the strongly structured parts of the filters. For filters that did not change across contrast conditions, the filter dissimilarity index yielded values near zero, whereas strong changes correspond to values close to unity.

#### Ganglion cell grouping for population analysis.

The analysis of filter changes at *locations X* and *Y* revealed considerable diversity of local and global adaptation effects across the population of ganglion cells in the mouse retina. To study whether the observed spatial adaptation patterns are related to other properties of the ganglion cells, we selected groups of cells that represented the most distinct adaptation patterns. Specifically, we distinguished four groups based on their filter dissimilarity values *FDI_X_* and *FDI_Y_* for *locations X* and *Y*, respectively: fixed-filter cells (*FDI_X_*<0.02, *FDI_Y_*<0.02), locally adaptive cells (*FDI_X_*>0.15, *FDI_Y_*<0.02), globally adaptive cells (*FDI_X_*>0.15, *FDI_Y_*>0.15), and translocally adaptive cells (*FDI_X_*<0.08, *FDI_Y_*>0.15). The thresholds in *FDI_X_* and *FDI_Y_* were chosen ad hoc, based on the population distribution of these values, so that each group contained ~20–30 samples. The grouping intends not to define specific types of cells, but rather to provide a basis for relating the spatial adaptation characteristics to other ganglion cell features. For the population analysis under stimulation with alternating contrast, for which overall fewer cells were recorded, the criterion for locally adaptive cells was slightly adjusted by requiring only *FDI_Y_*<0.08 to include more samples.

#### Analysis of nonlinearities.

To assess the sensitivity of a ganglion cell for each of the two stimulus components separately, we computed marginal nonlinearities, which estimate the relation of one stimulus component to the firing rate while averaging over the activation of the other stimulus component. Concretely, we first convolved the stimuli for *X* and *Y* with the respective temporal filters, as obtained from the spike-triggered average analysis and normalized to unit Euclidean norm. For each stimulus component, the marginal nonlinearity was then obtained as a histogram by binning the filtered signal into 40 bins, each containing approximately the same number of data points, and plotting, for each bin, the average filter signal against the average spike rate from the corresponding time points during the recording.

These marginal nonlinearities generally have a nonzero baseline, which is caused by spikes that were primarily triggered by the other stimulus component. This baseline therefore depends strongly on the contrast level at the other stimulus locations. For better comparing the shapes of the nonlinearities, we therefore shifted the marginal nonlinearities so that they all run approximately through the origin of the plot. This was achieved by subtracting the nonlinearity value at zero input, as obtained from a fitted sigmoidal function (see paragraph after next).

As a control, we also performed an alternative assessment of sensitivity by computing conditional nonlinearities (Garvert and Gollisch 2013; Samengo and Gollisch 2013), which aim at capturing the sensitivity to one stimulus component when the activation of the other stimulus component was near zero. Conditional nonlinearities were computed in the same fashion as marginal nonlinearities, except that, instead of taking all stimulus time points into account, we selected those time points when the filtered signal from the other stimulus component was inside a small range around zero. Concretely, when computing the conditional nonlinearity for *Y*, we chose time points when the filtered signal for *X* was between −0.3 × contrast and +0.3 × contrast, where “contrast” is the applied contrast level at *X* (1.0 or 0.2 for high or low contrast, respectively), and vice versa for the conditional nonlinearity for *X*.

To analyze sensitivity changes at the population level, we fitted the nonlinearities with a sigmoidal function, as used previously (Kastner and Baccus 2013):

$$r(s)=r_{0}+\frac{r_{\max}}{1+\exp\left(\frac{s_{1/2}-s}{a}\right)}$$

Here, *s* is the filtered stimulus, *r*_0_ is the basal firing rate, *r*_max_ is the maximal firing rate increase over the baseline, *s*_1/2_ is the location of the curve’s midpoint, and *a* is the steepness of the curve. *r*_0_, *r*_max_, *s*_1/2_, and *a* were optimized by a least-squares fit of *r*(*s*) to the histogram of the nonlinearity. From the fits, we calculated the sensitivity *S_LL_* for the low-low condition and the sensitivity *S_HL_* for the high-low condition as the slope values at *s*=0.3. This point of the curve was chosen because it lies approximately midway in the positive range of filtered signal values for the low-contrast condition where the nonlinearities are typically steep and not affected by threshold or saturation. Finally, we compared sensitivities by computing the ratio *S_LL_*/*S_HL_* for each of the two stimulus components *X* and *Y*. Using the slope of the nonlinearity here, rather than its maximal firing rate as we had done previously for salamander retinal ganglion cells (Garvert and Gollisch 2013), aims at measuring sensitivity independently of variations in the baseline activity, which we found to sometimes occur between contrast conditions in the mouse retina.

We also used the marginal nonlinearities to assess the degree of nonlinearity of individual cells. To do so, we compared the steepness of the nonlinearities for positive and negative inputs. Concretely, we used the nonlinearities of the low-low condition and extracted the slope values of the fitted sigmoid functions in the positive range (*S*_pos_, measured at *s*=0.3) and negative range (*S*_neg_, measured at *s*=−0.3). We then computed (*S*_pos_−*S*_neg_)/(*S*_pos_+*S*_neg_) and defined a cell’s nonlinearity index as the average of this value over *X* and *Y*. Fairly linear curves yield nonlinearity indices near zero, whereas nonlinearity indices near unity indicate a threshold-like nonlinearity.

#### Detection of image-recurrence-sensitive cells.

In a subset of experiments, we used shifting gratings to detect image-recurrence-sensitive (IRS) ganglion cells, as described previously (Krishnamoorthy et al. 2017). Briefly, a square-wave grating of 270-µm spatial period and 60% Michelson contrast was presented in a sequence of 800-ms-long fixations, separated by 100-ms transitions, during which the grating was shifted by approximately two spatial periods to land in one of four equally spaced fixation positions (corresponding to four specific spatial phases of the grating). The four fixation positions were numbered from 1 to 4, and their sequence was randomly chosen so that all 16 possible transitions between the starting position and the target position appeared randomly several times in the stimulus sequence.

We analyzed the responses by computing the peristimulus time histogram (PSTH) for each of the 16 transitions, using 10-ms bins. IRS cells were then identified as cells with sharp peaks in the PSTHs selectively for transitions where starting and target image were identical. Concretely, we computed a Recurrence Sensitivity Index in the following way (Krishnamoorthy et al. 2017): For the image recurrence of position *i* (i.e., where starting and target position of the grating were equal to *i*), we determined $D_{i}^{\text{(rec)}}$ as the maximal increase in the PSTH from one 10-ms bin to the next in the window from 50 to 250 ms after transition offset. Analogously, we determined $D_{i}^{\text{(change)}}$ as the maximal increase in the PSTH for the transition where the target position was equal to *i* and the starting position was the contrast-reversed grating. The Recurrence Sensitivity Index was then defined as $RSI=\frac{1}{4}\cdot \overset{4}{\underset{i=1}{\sum }}(D_{i}^{\text{(rec)}}-D_{i}^{\text{(change)}})\;/\;\left(D_{i}^{\text{(rec)}}+D_{i}^{\text{(change)}}\right)$, and IRS cells were determined as cells with RSI≥0.5.

#### Statistical testing.

To assess statistical significance of differences between contrast conditions or cell groups, we used nonparametric tests because Shapiro-Wilk tests indicated nonnormal distributions of several of the extracted parameters. We generally used a 5% significance criterion. Significance of contrast-induced changes of response parameters within a group of cells was assessed by a Wilcoxon signed-rank test. Significance of parameter differences between the four distinguished groups of cells were always first assessed by a Kruskal-Wallis test. We then further checked which specific cell groups were responsible for these parameter differences by performing a post hoc analysis of mean ranks with Bonferroni correction.

We graphically represented distributions of extracted parameters as box plots, which display the median and the interquartile range (IQR) from first to third quartile by a central line and a box, respectively. In addition, whiskers extend to most extreme values within 1.5×IQR, and outliers beyond this range are shown as distinct data points.

## RESULTS

### 

#### Diversity of spatial contrast adaptation.

**Fig. 2.**
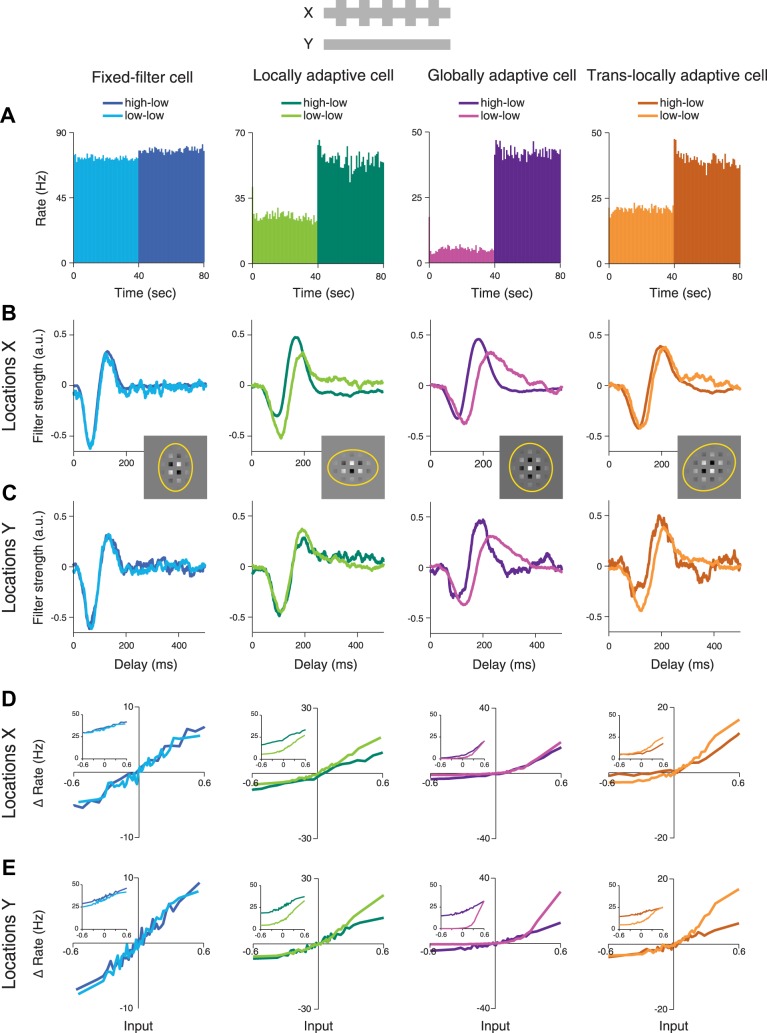
Filtering characteristics and nonlinearities of 4 sample cells, corresponding to the 4 distinguished groups of cells, comparing activity under the high-low contrast condition (dark color shades) and low-low condition (brighter shades). *A*: firing rate histograms averaged over all trials during the low-low condition (0–40 s) and the high-low condition (40–80 s). *B*: filters obtained for *locations X*. *Insets* show how the stimulus relates to the receptive fields by displaying white and black squares for *locations X* and *Y*, respectively, and weighting the contrast by the Gaussian fit to the receptive field of the corresponding cell. The yellow ellipses display the 3-σ contour lines of the Gaussian fits. *C*: filters obtained for *locations Y*. *D*: marginal nonlinearities obtained for *locations X*, shown over the range spanned by the low contrast stimulation and shifted to pass approximately through the origin (see methods) so that responses are measured as changes in firing rate (“Δ Rate”). *Insets* show the nonshifted nonlinearities. *E*: marginal nonlinearities obtained for *locations Y*.

We studied the spatial scope of contrast adaptation in ganglion cells of isolated mouse retina by recording spikes with a stimulus that contained local changes in contrast. The stimulus consisted of two sets of regions, called *locations X* and *locations Y*, respectively, which were simultaneously stimulated with independent white-noise sequences of light intensity. The range of light intensities in the applied sequence determines the stimulus contrast level, here defined as the standard deviation of light intensities relative to their mean. For *locations X*, this stimulus contrast switched every 40 s between a low level (20% contrast) and a high level (100%), whereas contrast remained fixed at 20% for *locations Y* (Fig. 1). For each cell, contrast-induced changes in temporal filtering and sensitivity were analyzed separately for the two sets of locations by reverse-correlation analysis that provided separate temporal filters and marginal nonlinearities (see methods). Figure 2 shows exemplary results from four ganglion cells that are representative of different types of adaptation characteristics. As expected, the cells increased their firing rates when stimulation was switched from low contrast at both sets of locations (low-low condition, Fig. 2*A*, 0–40 s) to high contrast at *X* and low contrast at *Y* (high-low condition, Fig. 2*A*, 40–80 s).

Effects of contrast on temporal filtering were assessed by computing the spike-triggered average for each of the two contrast conditions and separating it into a temporal filter for locations where contrast changed (*locations X*, Fig. 2*B*) and a temporal filter for locations where contrast remained constant (*locations Y*, Fig. 2*C*). For the first sample cell, both temporal filters remained largely unchanged across the two contrast conditions, indicating that there were hardly any contrast-induced changes in temporal filtering for this cell. For convenience during further analysis, we termed cells with this behavior fixed-filter cells. The second sample cell showed a marked change for the filter at *X* with faster kinetics and a more biphasic shape, but an essentially unchanged filter at *Y*. This suggests that changes in temporal filtering were locally confined to regions inside the receptive field where contrast actually changed. Cells with this behavior are in the following called locally adaptive cells. By contrast, the third sample cell displayed pronounced, nearly identical filter changes at both *X* and *Y*, indicating that local changes in contrast affected the entire receptive field of this cell in a virtually uniform fashion. We call cells with such behavior globally adaptive cells. Finally, the fourth sample cell is representative of a subset of cells for which filter shapes were affected for both sets of locations, yet the changes were more pronounced at *locations Y*. This means that the effects of contrast adaptation on temporal filtering were actually more pronounced at locations where contrast stayed constant, and we therefore call these cells translocally adaptive cells.

We also investigated how the local contrast changes at *locations X* affected the sensitivity of the cells for stimuli at either *locations X* or *locations Y*. To do so, we computed marginal nonlinearities for both sets of locations separately (see methods), which capture how, on average, the ganglion cell translated activation at the corresponding location into a firing rate (Fig. 2, *D* and *E*). To focus on comparing the shape and steepness of the nonlinearities as a measure of sensitivity, we shifted them to approximately pass through the origin of the plots. This was done because the marginal nonlinearities generally contain an elevated baseline, which is caused by spikes generated from activation at the other set of stimulus locations and which therefore depends on the contrast level at the other set of locations. The nonshifted marginal nonlinearities are shown as insets in Fig. 2, *D* and *E*. Except for the fixed-filter cell, the sample cells showed changes in the nonlinearity at both sets of locations. In particular, for strong positive activation, nonlinearities for the low-low condition were considerably steeper, indicative of a higher gain. These sensitivity changes occurred not only at locations where contrast changed, but also for locations where contrast remained constant, indicative of at least some degree of global sensitivity adaptation after local contrast changes.

#### Quantification of filter changes and grouping of cells into adaptation classes.

The four sample cells of Fig. 2 showed that different ganglion cells can display quite different spatial transfer properties of adaptation effects evoked by local contrast changes. To systematically assess the local and global contributions to contrast-induced changes in temporal filtering and sensitivity over the population of recorded ganglion cells, we aimed at quantifying the filter and sensitivity changes, while distinguishing between different adaptation patterns as displayed by the four examples of Fig. 2. We therefore sought a simple approach for grouping cells according to their adaptation characteristics by computing a filter dissimilarity index to quantify differences in filter shape between the low-low and high-low contrast conditions (see methods). Filter dissimilarity indices near zero correspond to constant filter shapes across contrast condition, whereas indices near unity indicate strong filter changes.

**Fig. 3.**
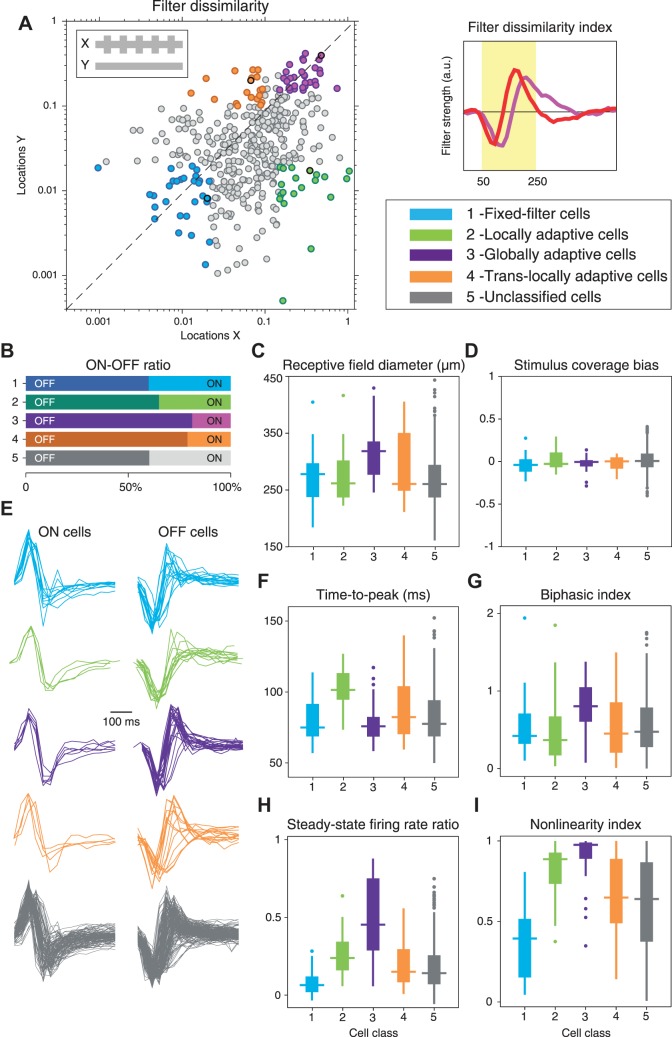
Characterization of cell classes with differences in the spatial scope of contrast adaptation. *A*: quantification of filter dissimilarity at *locations X* and *Y* for all recorded cells (*n* = 398). The filter dissimilarity values provided the basis for selecting 4 groups of cells, as indicated by the colors, to represent the cases with strong filter changes at *X*, at *Y*, at both, or at neither set of locations. The 4 sample cells of Fig. 2 are marked by thick black outlines. On the right, the shaded area behind the displayed sample filters marks the temporal window used for computing the filter dissimilarities. *B*: distributions of ON vs. OFF cells for the 4 groups. *C*: distributions of receptive field sizes. *D*: distributions of stimulus coverage bias. *E*: temporal filters, obtained from spatiotemporal white-noise stimulation, separated into the 4 groups and into ON and OFF cells. *F*: distributions of time-to-peak values obtained from the filters shown in *E*. *G*: distributions of biphasic indices obtained from the filters shown in the *E*. *H*: distributions of the ratio of the steady-state firing rate between the 2 contrast conditions. *I*: distributions of nonlinearity indices.

Plotting the filter dissimilarity indices for both *locations X* and *locations Y* for all recorded cells (Fig. 3*A*) shows a continuum of quantitative filter changes, with some cells having large filter dissimilarity indices at *X* as well as at *Y* (globally adaptive cells), some cells having small filter dissimilarity indices at both locations (fixed-filter cells), and some cells having considerably larger filter dissimilarity indices at either *X* or *Y* as compared with the other location (locally adaptive cells and translocally adaptive cells, respectively). Although the continuous distribution of the data did not suggest distinct adaptation types, we used it to group cells into rough classes to further analyze how the different general adaptation characteristics relate to other response features. We thus selected four groups of sample cells along the edges of this distribution to represent the four combinations with changing or constant filters at *X* and *Y* (Fig. 3*A*, see also methods). Note that this represents merely a pragmatic choice to facilitate further population analysis, and we do not claim that the groups capture discrete categories of adaptation or individual cell types, nor that four would be the right number of groups to consider. Correspondingly, the labels that we attach to these groups, such as “globally adaptive cells,” are not meant to imply a distinctive type of cells but are rather used as a shorthand for referring to cells that primarily showed, for example, global adaptation characteristics.

#### Differences in general response properties between the adaptation classes.

We used the obtained grouping of cells to first check whether the distinguished adaptation classes also differed in their preferred contrast polarity. Based on the temporal component of the receptive field, we classified each cell as an ON or OFF cell and found that both response types were well represented in each of the distinguished adaptation groups (Fig. 3*B*). Thus, both ON and OFF ganglion cells showed similar diversity of the spatial scope of contrast adaptation, which was therefore not directly linked to differences in the preferred contrast polarity. By contrast, when considering the size of the spatial receptive field (Fig. 3*C*), we found that globally adaptive cells were comparatively large (average diameters of 314 ± 45 µm, denoting mean ± SD as in all subsequent quantification of population data) and differed significantly (*P* = 0.005, Kruskal-Wallis test) from the fixed-filter and locally adaptive cells (fixed-filter cells: 272 ± 48 µm; locally adaptive cells: 278 ± 51 µm). Translocally adaptive cells showed no clear difference from other groups of cells (287 ± 59 µm). Given these differences in receptive field size, we checked whether there was any systematic difference between the groups of cells in how the stimulus components covered the receptive fields. Analysis of the stimulus coverage bias (cf. Fig. 1*C*), however, showed that for all four groups, both stimulus components contributed nearly equally to the receptive field coverage (Fig. 3*D*), without significant differences between the groups (*P* = 0.72, Kruskal-Wallis test).

Locally adaptive and globally adaptive cells not only differed in their receptive field sizes but also showed clear differences in the temporal profiles of the receptive fields. Figure 3*E* shows the extracted temporal filters for the selected sample cells according to the four groups. Most strikingly, globally adaptive cells with few exceptions displayed short time-to-peak values and particularly biphasic filters (Fig. 3, *F* and *G*), suggesting that the global scope of contrast adaptation is primarily found for fast, transient ganglion cell types. By contrast, locally adaptive cells were characterized by rather slow, monophasic filter shapes (Fig. 3*G*) and were thus distinct from globally adaptive cells in their temporal filtering characteristics. Fixed-filter cells typically showed fast filters with time-to-peak comparable to globally adaptive cells, but with only mild biphasicness, comparable to locally adaptive cells (Fig. 3*G*). Finally, translocally adaptive cells showed a wide range of filter shapes with no characteristic pattern.

The original four sample cells had varied strongly in the contrast-dependent changes of the activity level (Fig. 2*A*). Average firing rates changed dramatically across contrast conditions for the globally adaptive cell, but only little for the fixed-filter cell. To check whether this is a systematic effect, we computed the normalized difference in steady-state firing rates between the high-low and the low-low condition (see methods). Indeed, globally adaptive cells typically had much stronger changes in average firing rates than the other groups (*P* < 10^−11^, Kruskal-Wallis test), whereas fixed-filter cells only displayed small differences in average activity between the low-low and the high-low contrast condition (Fig. 3*H*). Locally adaptive cells showed intermediate contrast-induced changes of the activity level, which were more pronounced than for fixed-filter cells (*P* < 10^−4^), but smaller than for globally adaptive cells (*P* < 10^−4^).

These differences in the contrast-induced changes of the average firing rate may depend on how nonlinear the cells are in their light responses. The fixed-filter cell of Fig. 2, for example, had a rather linear response relation so that firing rate elevations and reductions under higher contrast may approximately balance each other out. The globally adaptive cell of Fig. 2, on the other hand, displayed a strong, threshold-like nonlinearity, so that higher contrast can lead to a net increase in average firing rate. Indeed, we found that globally adaptive cells generally had more pronounced nonlinearities than the other groups (*P* < 10^−4^ in all cases), as quantified by a nonlinearity index that compares the slopes of the marginal nonlinearities for positive and negative input (Fig. 3*I*). Conversely, fixed-filter cells generally had much less pronounced nonlinearities, with smaller nonlinearity indices than cells from the other groups (*P* < 10^−4^ in all cases).

Together, these analyses suggest that the different patterns of spatial contrast adaptation depend on cell type, yet likely span multiple cell types each, e.g., ON-type and OFF-type cells. Most globally adaptive cells had large receptive fields, strongly biphasic filters, pronounced nonlinearities, and a strong dependence of the average activity on contrast level. Locally adaptive cells, on the other hand, were typically small with slow, monophasic temporal filters. Fixed-filter cells often had also small receptive fields and rather monophasic temporal filters and, in particular, showed the most linear dependence of firing rate on activation level and the smallest changes of average firing rates across contrast levels. Finally, translocally adaptive cells were the least homogeneous group with generally intermediate response characteristics.

**Fig. 4.**
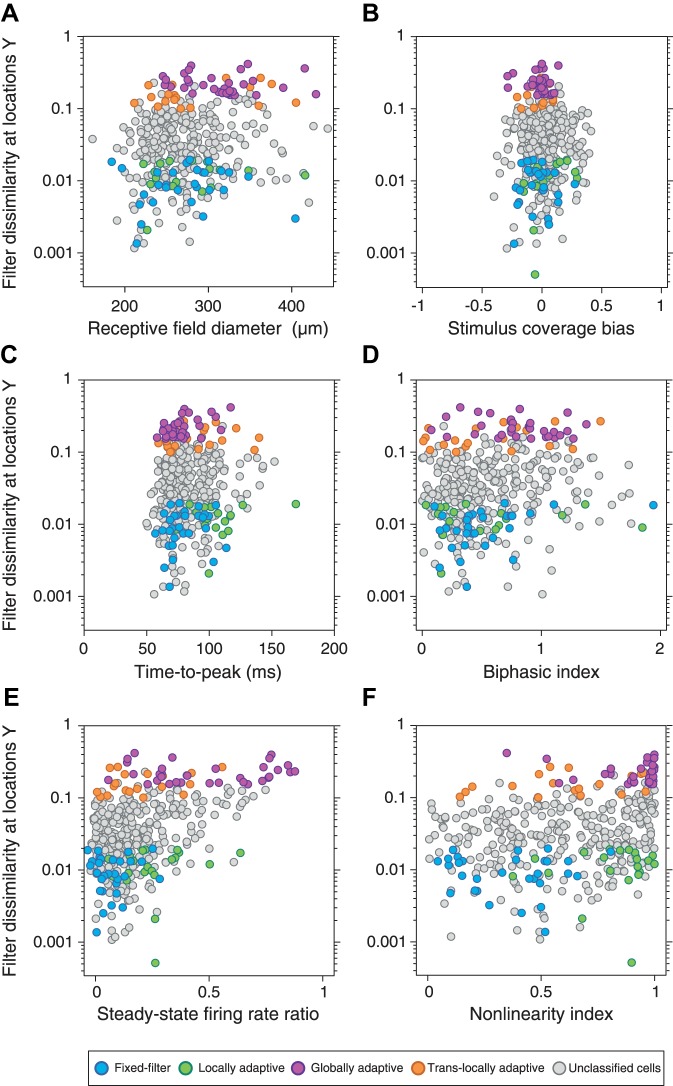
Cell-by-cell analysis of how contrast-induced filter changes at *locations Y* relate to basic response characteristics. *A*: relation to receptive field size. *B*: relation to stimulus coverage bias. *C*: relation to time-to-peak of the filters, as obtained in Fig. 3*F*. *D*: relation to filter biphasicness. *E*: relation to the ratio of the steady-state firing rate between the 2 contrast conditions. *F*: relation to the nonlinearity index, which quantifies how nonlinear the cells’ responses are relative to their filter activation. Colors indicate cells of the selected groups, as defined in Fig. 3*A*.

The selection of four groups of ganglion cells aims at providing structure to the observed variability of spatial adaptation profiles by focusing on the most pronounced adaptation patterns. Yet, the continuous distribution of filter changes (Fig. 3*A*) may suggest handling this variability in a continuous fashion by correlating the dissimilarity of the filters under different contrast conditions to other response features. This would also allow taking all recorded cells into account and thereby serve to test whether focusing on cells from the edges of the distribution of filter changes gives a skewed picture, not representative of the overall population of cells. Thus, to check whether our selection of groups biased our analysis towards cells with particular response properties, we analyzed basic response characteristics for all cells to relate them to the filter changes on a cell-by-cell basis. An example is shown in Fig. 4, which displays scatter plots of the filter changes at *Y* (where contrast stayed constant), depending on the various response characteristics of the cells.

The plots primarily indicate that there is no simple relation between general response properties and filter changes beyond what could be extracted from Fig. 3. The data form a continuum with relation to the investigated response characteristics, and the selected four groups represent the spread of the data. For example, for the relation to the change in steady-state firing rate (Fig. 4*E*), it becomes apparent that strong firing rate changes nearly always induce filter changes at *Y*, as mostly captured by the group of globally adaptive cells. However, the plots also show that focusing on filter changes at only one set of locations (here *Y*) does not give the full picture. In Fig. 4*E*, for example, this analysis does not by itself distinguish between globally adaptive cells, which do display a strong change in firing rate, and translocally adaptive cells, which do not show such a strong change in firing rate, but still have a strong filter change for *locations Y*; this structure is only revealed by simultaneously considering filter changes also at *locations X*. Similarly, locally adaptive cells display a large time-to-peak (Fig. 4*C*) and a large nonlinearity index (Fig. 4*F*), yet, on the population level, this is counteracted by other cells, in particular fixed-filter cells, that also have small filter dissimilarity for *locations Y* but, unlike locally adaptive cells, do not have strong filter changes for *locations X*. Thus, for a clearer picture, we need to simultaneously take into account the filter changes at both *X* and *Y*, and the grouping of cells as in Fig. 3 is a straightforward way to do so. For further analysis, we therefore focus on the selected groups because they appear to reasonably represent the distribution of adaptive filter changes and because they allow us to simultaneously take the effects at both sets of locations into account.

#### Spatial scope of contrast adaptation for a specific ganglion cell type.

**Fig. 5.**
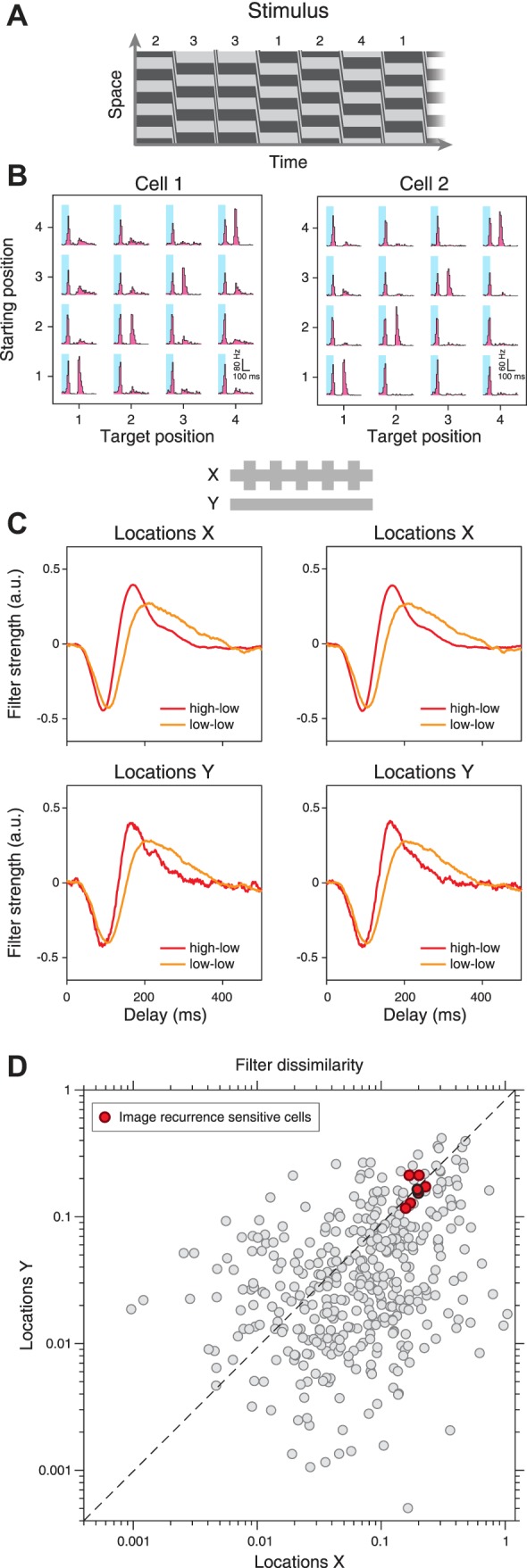
Image-recurrence-sensitive (IRS) cells consistently display global contrast adaptation. *A*: schematic spatiotemporal display of the stimulus used for identifying IRS cells. The numbers on top indicate fixation positions of the grating, which are separated by rapid shifts of the grating. *B*: matrix of PSTHs for all 16 possible transitions between different starting positions (*y*-axis) and target positions (*x*-axis) for 2 sample cells, displaying the characteristic second response peak for transitions with equal starting and target position. The shaded regions mark the 100-ms transition periods. *C*: temporal filters obtained for the same 2 cells as in *B* for the contrast-adaptation stimulus that switched between the low-low condition (light orange) and the high-low condition (dark orange). *D*: scatter plot of filter dissimilarity indices for all recorded cells (same data as in Fig. 3*A*) with all identified IRS cells (*n* = 7) highlighted in red. The 2 sample cells are indicated by thick black outlines.

Before analyzing the adaptation characteristics of the four classes of cells further, however, we aimed at testing the idea that the type of filter changes depend on cell type. Distinguishing the more than 30 different types of ganglion cells based on functional characterizations is not an easy task (Baden et al. 2016), yet some specific cell types may be detected through their characteristic responses to particular stimulus patterns. Thus, to check how a specific ganglion cell type relates to the broad continuum of local and global contrast adaptation effects, we aimed at identifying image-recurrence-sensitive (IRS) cells in some of our experiments. These cells show a characteristic sensitivity to the recurrence of an image under saccade-like transitions, and they correspond to the specific type of transient OFF-alpha ganglion cells (Krishnamoorthy et al. 2017). We therefore stimulated the retina with a square-wave grating that was repeatedly shifted between four different fixation positions (Fig. 5*A*). We then identified IRS cells by their posttransition response peaks, which occurred selectively for those transitions where the grating positions before and after the transition were identical (Fig. 5*B*, see methods). When checking the changes in temporal filtering for these cells, we found that all identified IRS cells consistently displayed changes in the filters for *locations X* as well as for *locations Y*, in a way that was remarkably similar between individual cells (Fig. 5, *C* and *D*), with apparent global adaptation characteristics. This corroborates our conclusion that the spatial scope of contrast adaptation is a cell-type specific feature.

#### Characteristics of contrast-induced filter changes.

**Fig. 6.**
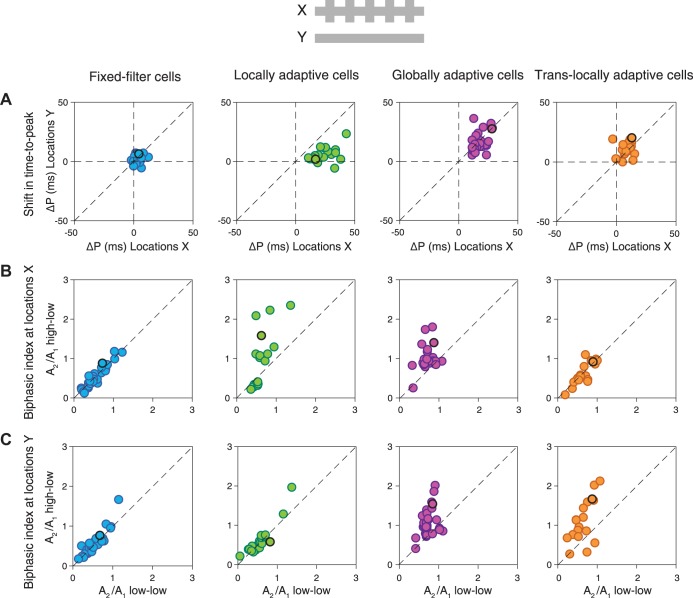
Population analysis of filter changes in response to local contrast changes. *A*: shift in time-to-peak (Δ*P*) of the filters between the low-low and high-low conditions, compared for *locations X* and *locations Y*. Here and in subsequent panels, the dashed diagonal line indicates identity. The circles with dark edges mark the data from the 4 sample cells of Fig. 2. *B*: biphasic indices (computed as the ratio *A*_2_/*A*_1_ of the secondary and primary filter peak amplitudes) for *locations X*, compared for the low-low and high-low conditions. *C*: biphasic indices for *locations Y*. (Fixed-filter cells: *n* = 30; locally adaptive cells: *n* = 20, globally adaptive cells: *n* = 32; translocally adaptive cells: *n* = 19.)

To better characterize the nature of the contrast-induced filter changes for the four selected classes of ganglion cells, we quantified the shift in time-to-peak between the contrast conditions (Fig. 6*A*) and compared the biphasicness for the two conditions at *locations X* (Fig. 6*B*) as well as at *locations Y* (Fig. 6*C*). As expected, fixed-filter cells had no or very little shift in time-to-peak at either *X* or *Y* (*X*: 3 ± 3 ms; *Y*: 2 ± 3 ms), and their biphasicness remained approximately unaltered for both *X* and *Y*. For locally adaptive cells, the local scope of changes in temporal filtering was apparent in both the peak time and the biphasicness, indicating that these cells are locally adaptive both in the kinetics and in the frequency selectivity. The cells displayed large shifts in time-to-peak for *locations X* (23 ± 9 ms), but not for *Y* (6 ± 5 ms), and their biphasicness was strongly increased for the high-low condition at *locations X* (high-low: 1.20 ± 1.01; low-low: 0.61 ± 0.28; *P* = 0.01), but not at *Y* (high-low: 0.63 ± 0.39; low-low: 0.57 ± 0.39; *P* = 0.1). By contrast, globally adaptive cells showed large shifts in time-to-peak for both *X* and *Y* (X: 18 ± 5 ms; Y: 17 ± 6 ms), with no significant difference between *locations X* and *Y* (*P* = 0.2). Biphasic indices of globally adaptive cells generally increased for both locations during the high-low condition, though some cells also showed no change in biphasicness. For translocally adaptive cells, the most striking feature was that biphasicness changed strongly and significantly for *locations Y* (high-low: 1.20 ± 0.51; low-low: 0.64 ± 0.24; *P* = 0.001), but not for *locations X* (high-low: 0.61 ± 0.31; low-low: 0.65 ± 0.24; *P* = 0.15). This shows that the peculiar nonlocal adaptation of temporal filtering in these cells corresponds to altered local frequency selectivity rather than altered kinetics.

#### Adaptation of local sensitivity.

Adapting to new contrast not only affects temporal filtering in ganglion cells but also changes their sensitivity (Baccus and Meister 2002; Demb 2008; Kim and Rieke 2001). To measure the changes in local sensitivity for each of the two *locations X* and *Y* separately, we analyzed the marginal nonlinearities that measure sensitivity at each of the two locations. When contrast at *locations X* increased, the marginal nonlinearities for both sets of locations changed in shape (Fig. 2, *D* and *E*). In particular, we typically observed increased steepness of the nonlinearities for positive activation during the low-low contrast condition, reflecting a larger gain at lower contrast level.

**Fig. 7.**
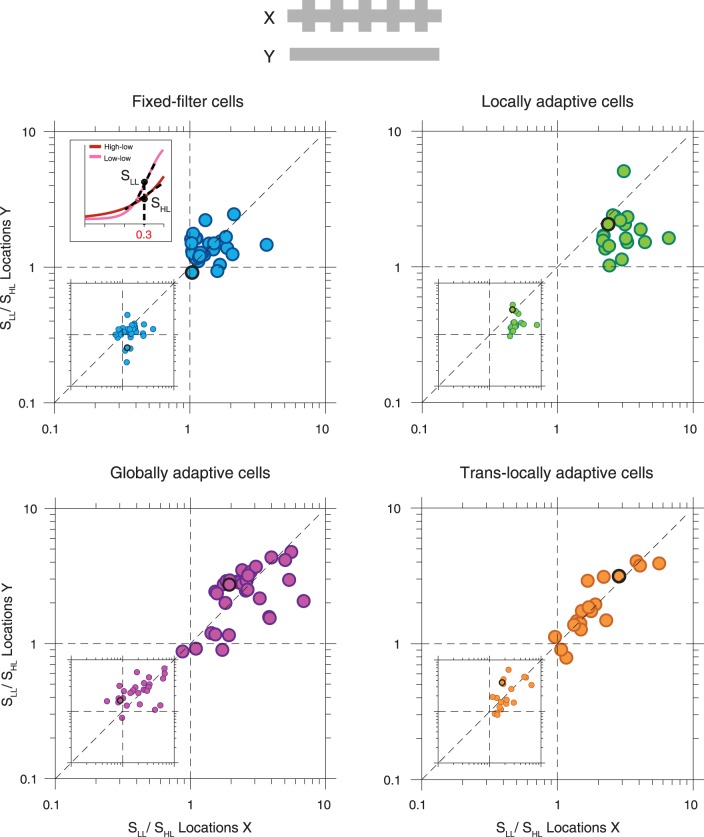
Population analysis of the changes in sensitivity in response to local contrast changes, measured as the ratio of the sensitivities under low-low (*S_LL_*) and high-low (*S_HL_*) contrast conditions. Sensitivity was calculated, as depicted in the inset in the upper-left corner, as the slope of the fitted marginal nonlinearity at an input value of 0.3. As a control, *insets* in the lower-left corners show the same sensitivity measure, but computed from conditional nonlinearities, obtained by selecting time points for which the other stimulus component had filter activation near zero (see methods). The conditional nonlinearities confirm the results obtained with marginal nonlinearities, albeit with higher noise level because the conditional nonlinearities are based on restricted subsets of the data.

To quantify the contrast-induced changes in sensitivity, we here measured the gain as the steepness of the marginal nonlinearity for positive filter output (Fig. 7). Specifically, we fitted the nonlinearity with a sigmoidal function and defined the sensitivity measures S_LL_ of the low-low condition and S_HL_ of the high-low condition by the slope of the corresponding function at a fixed position (see methods). We compared sensitivities for the two contrast conditions by calculating the ratio S_LL_/S_HL_ for both *X* and *Y*. Values close to unity correspond to similar sensitivity for both contrast conditions, and larger values indicate higher sensitivity during the low-low condition.

The data show that most analyzed cells had higher sensitivity during the low-low condition for both *X* and *Y*, indicative of at least some global adaptation of sensitivity. For fixed-filter cells, the sensitivity changes were smaller than for the other three groups of cells. For these cells as well as for globally adaptive and translocally adaptive cells, the sensitivity ratio S_LL_/S_HL_ showed no significant difference between the two locations (*P* > 0.4 in all cases), suggesting that sensitivity adapts primarily in a global fashion under contrast changes for these three groups of cells. By contrast, locally adaptive cells displayed much larger sensitivity ratios for *locations X* than for *locations Y* (*P* < 10^−3^). Thus, locally adaptive cells showed a local spatial scope of contrast adaptation not only for their temporal filters, but also for their sensitivity.

#### Local contrast adaptation under constant global contrast.

**Fig. 8.**
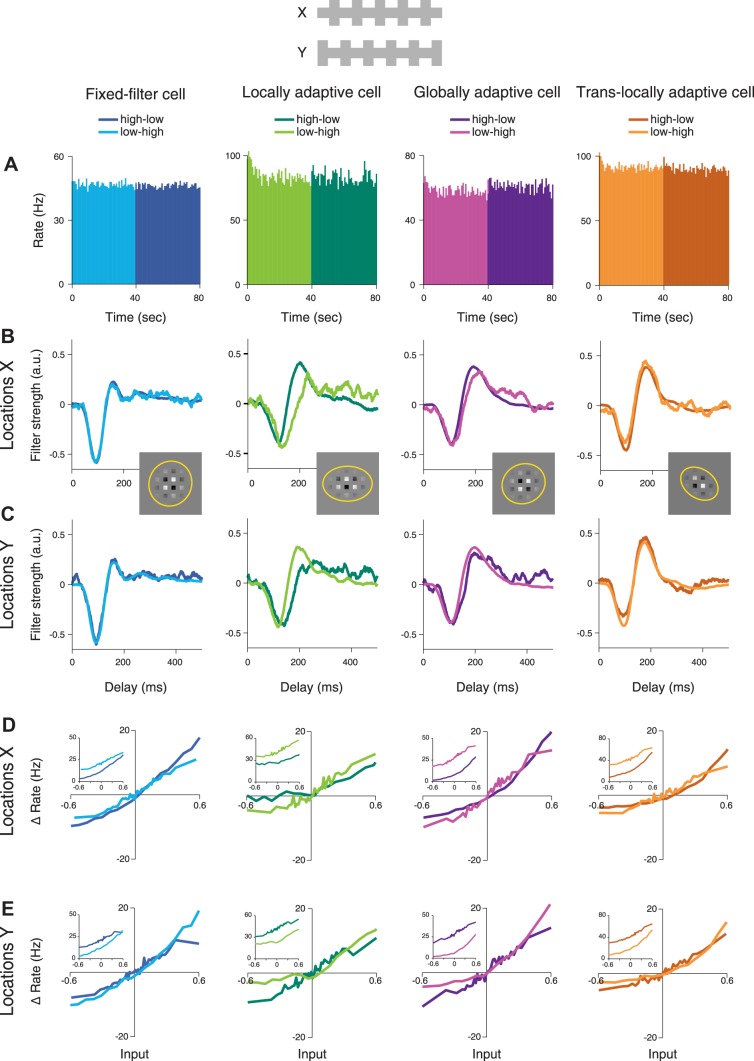
Filtering characteristics and nonlinearities of ganglion cells under local contrast changes with constant global contrast, comparing activity under the high-low contrast condition (high contrast at *X*, low contrast at *Y*, dark color shades) and the low-high condition (low contrast at *X*, high contrast at *Y*, brighter shades). *A*: firing rate histograms, averaged over all trials during the low-high condition (0–40 s) and the high-low condition (40–80 s). *B*: filters obtained for *locations X*. *C*: filters obtained for *locations Y*. *Insets* display the relation of the stimulus to the receptive fields as in Fig. 2*B*. *D*: marginal nonlinearities obtained for *locations X*, over the range spanned by the low contrast stimulation, shifted to pass approximately through the origin of the plot so that responses are measured as changes in firing rate (“Δ Rate”). Insets show nonshifted nonlinearities. *E*: marginal nonlinearities obtained for *locations Y*.

Contrast-adaptation effects that occur on a global spatial scope should be reduced when local contrast changes occur in such a fashion that global contrast over the receptive field remains constant. Local contrast adaptation effects, on the other hand, should then prevail. To test this scenario, we used a stimulus that contained alternating high-contrast episodes at *locations X* and *locations Y*, thus switching between a high-low (high contrast at *X*) and a low-high (high contrast at *Y*) condition. Thus, while the previous stimulus contained both global and local contrast changes, the stimulus considered now aimed at keeping global contrast constant while still changing local contrast. Figure 8 shows results from four sample cells, representing the four distinguished adaptation groups.

We observed that this stimulus indeed kept global contrast approximately constant for each cell because the average firing rates were about equal for the high-low and low-high contrast conditions (Fig. 8*A*). Nonetheless, the locally adaptive cell showed strong filter changes between the contrast conditions, whereas filters were more similar across contrast conditions for the other sample cells. This corroborates the strongly local scope of contrast adaptation in cells that we identified as locally adaptive. Furthermore, the translocally adaptive cell had slightly more biphasic filters for either *X* or *Y* whenever contrast was high at the other set of locations, although apart from a small difference in amplitude of the first filter peak, the filters look nearly identical for the two contrast conditions. This underscores that the translocal nature of contrast-induced filter changes is restricted to changes in biphasicness. Finally, this stimulus revealed a small local adaptation component in the biphasicness of the filters for the globally adaptive cell. Note that the effect here was that biphasicness was larger for locations where contrast was high, which is opposite to the effect observed for the translocally adaptive cell.

**Fig. 9.**
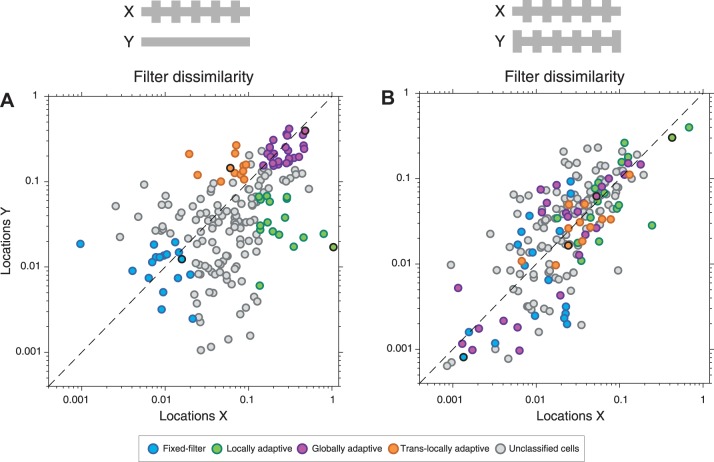
Comparison of filter dissimilarity under local contrast changes with and without changes in global contrast. *A*: filter dissimilarity indices under the standard stimulus with contrast changes restricted to *locations X*. The plot only shows cells that were also recorded under stimulation with high contrast alternating between *locations X* and *Y* (*n* = 193), and the data therefore constitute a subset of the data shown in Fig. 3*A*. *B*: filter dissimilarity indices for the same cells as in *A*, obtained under stimulation with high contrast alternating between *locations X* and *Y*. Colors indicate cells of the 4 distinguished adaptation groups, as defined according to their filter dissimilarity values displayed in *A*. The 4 sample cells of Fig. 8 are marked by thick black outlines.

**Fig. 10.**
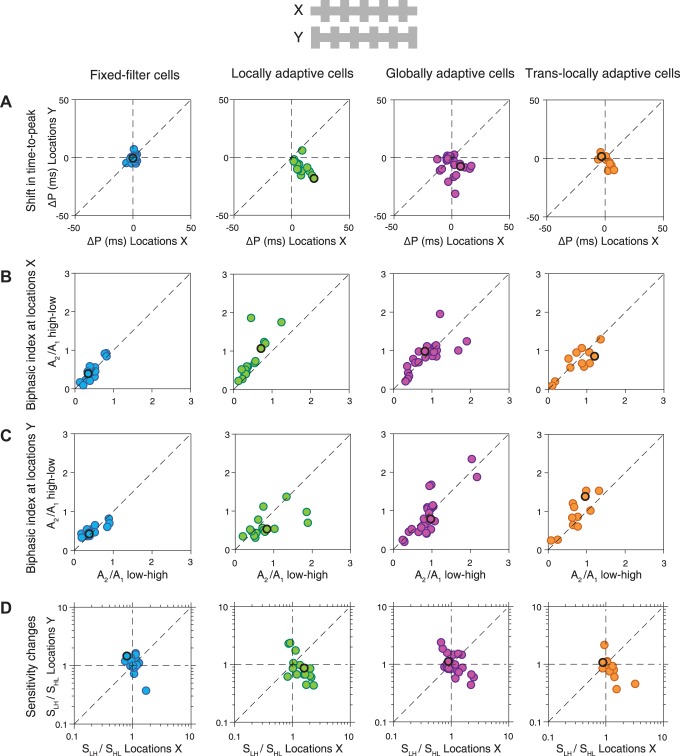
Population analysis of filter and sensitivity changes in response to local contrast changes with constant global contrast. *A*: shifts in time-to-peak of the filters between the high-low and low-high conditions, compared for *X* and *Y*. *B*: biphasic indices for *locations X*, compared between the high-low and low-high conditions. *C*: same as *B*, but for *locations Y*. *D*: sensitivity changes, compared between *X* and *Y*. The 4 sample cells of Fig. 8 are marked by thick black outlines. **(**Fixed-filter cells: *n* = 17; locally adaptive cells: *n* = 16; globally adaptive cells: *n* = 25: translocally adaptive cells: *n* = 12.)

Population analysis confirmed these observations. First, we computed the filter dissimilarity indices for the original stimulus (Fig. 9*A*) and for the new stimulus with alternating high-contrast locations (Fig. 9*B*) for all cells recorded under this new stimulus. Note that for the latter, contrast changed at both sets of locations so that local adaptation here led to high filter dissimilarity for both *X* and *Y*. This was observed primarily for locally adaptive cells, which provided data points primarily to the upper right of Fig. 9*B*. Globally adaptive cells, on the other hand, now often had small filter dissimilarity for both *X* and *Y*, indicating that adaptation effects were reduced in these cells when local contrast changed, but global contrast stayed constant. For a clearer picture of how filters and sensitivity changed under local contrast changes with constant global contrast, we again analyzed the changes separately for each of the four distinguished groups (Fig. 10).

Changes in time-to-peak (Fig. 10*A*) were most systematic for locally adaptive cells, with earlier time-to-peak whenever the contrast was high at the corresponding location (mean changes in time-to-peak, here defined as P_low-high_ – P_high-low_, at *X*: 8.3 ± 5.5 ms, *P* < 10^−3^; at *Y*: −7.7 ± 6.5 ms, *P* < 10^−3^). Cells from the other groups showed no or small shifts in time-to-peak; only globally adaptive cells displayed a significant shift for *locations Y* (−6.9 ± 7.4 ms, *P* < 10^−4^), though not for *locations X*. Locally adaptive cells furthermore showed systematic effects also in the biphasicness, with generally more biphasic filters when the corresponding locations experienced high contrast, though this effect was significant only for *locations X* (Fig. 10, *B* and *C*; *P* < 10^−4^ for *X* and *P* = 0.08 for *Y*).

Finally, the sensitivity changes under this stimulus paradigm were often small, with sensitivity ratios near unity (Fig. 10*D*), and generally smaller than for the previous stimulus paradigm (cf. Fig. 7). This is consistent with the mostly global scope of contrast-induced sensitivity changes observed above because the present stimulus keeps global contrast approximately constant and therefore does not effectively trigger global mechanisms of sensitivity adaptation. Local sensitivity changes, on the other hand, could still be triggered and should reveal themselves in opposing sensitivity changes for *locations X* and *Y*, that is, data points along the lower-right diagonal in Fig. 10*D*. This can be observed for the locally adaptive cells and was corroborated by a statistical analysis: Since contrast was low at *X* during the low-high condition and low at *Y* during the high-low condition, we tested whether *S_LH_*/*S_HL_* for *locations X* and *S_HL_*/*S_LH_* for *locations Y* were systematically larger than unity. This was the case for locally adaptive cells (*P* = 0.0021, sign test). Translocally adaptive cells also showed a number of data points along the lower-left diagonal, but this was not quite significant in our data (*P* = 0.064, sign test). Fixed-filter and globally adaptive cells did not reveal any local sensitivity adaptation here (*P* = 0.87 and *P* = 0.32, respectively). Thus, again, locally adaptive cells display local adaptation in sensitivity most clearly, confirming that these cells are locally adaptive not only in their temporal filtering properties, but also in their sensitivity.

## DISCUSSION

The visual system adjusts its stimulus processing characteristics to changes in visual contrast. Often, this contrast adaptation is investigated by considering spatially homogeneous contrast changes that affect the entire visual scene. Yet, natural scenes are likely to contain local variations in contrast, owing to different objects, textures, or lighting conditions in the scene. Thus, changes in contrast may often occur in a local fashion, for example, when objects move or the gaze direction shifts.

We here investigated whether such local changes in contrast, when they occur on a scale smaller than a receptive field, lead to local adaptation effects, confined to the region where contrast changed, or whether they can affect the receptive field in its entirety. Whether contrast adaptation occurs locally or globally over receptive fields is important for both ganglion cell function and for understanding mechanisms of contrast adaptation (Brown and Masland 2001; Garvert and Gollisch 2013; Rieke and Rudd 2009). Local adaptation of sensitivity allows a ganglion cell, for example, to remain sensitive to low-contrast features even when other parts of the receptive field are stimulated at high contrast, providing for a locally adjusted integration of spatial information across the receptive field. Global adaptation, on the other hand, focuses a cell’s stimulus encoding potential on the features of highest contrast in the receptive field, at the expense of weaker contrast signals at other parts of the receptive field, much like a winner-take-all representation. The difference between local and global sensitivity changes appears particularly relevant for moving objects that cross the receptive field. In the case of global adaptation, the entry of the object into the receptive field will be more strongly represented than its subsequent trajectory through the adapted receptive field, which has been suggested as an important contribution to motion anticipation phenomena (Berry et al. 1999; Chen et al. 2014; Leonardo and Meister 2013).

### 

#### Cell-specific differences in spatial scope of contrast adaptation in mouse retina.

For ganglion cells of mouse retina, we here found a surprising diversity, ranging from no or very small contrast adaptation effects to pronounced local or global contrast adaptation. This is in striking contrast to our previous observations in the salamander retina (Garvert and Gollisch 2013) where adaptation phenomena appeared much more homogeneous and where global adaptation predominated, with most recorded cells appearing similar to what we here consider as globally adaptive cells. We hypothesize that the spatial scope of contrast adaptation observed in mouse retina is cell-type specific. This is supported by two observations. First, we found that the contrast-induced changes in temporal filtering are related to other response features of the cells. For example, globally adaptive cells tend to have large receptive fields, whereas locally adaptive cells have slow filters, and fixed-filter cells are the most linear in their contrast encoding (Fig. 3, *C*–*I*). And second, for one particular type of cells, namely transient OFF-alpha cells, which were identified via their sensitivity to image recurrence across saccade-like image transitions (Krishnamoorthy et al. 2017), we could indeed show that the adaptation characteristics are quite similar for different cells of the same type.

Nonetheless, we cannot exclude that, for individual cells, the observed contrast-induced changes at the two sets of locations are also influenced by the particular placement of the stimulus subfields onto the cells’ receptive fields, for example, because a given cell might be more sensitive to stimuli either at *locations X* or at *locations Y*. Yet, it seems unlikely that the stimulus placement has a strong effect on our general findings regarding the observed diversity and the distinguished types of spatial scope in contrast adaptation. First, cells were generally about equally sensitive to both stimulus components, as measured by the stimulus coverage bias (Fig. 1*C*), and each of the four adaptation groups contained examples with slightly more sensitivity for *X* as well as for *Y* (Figs. 3*D* and 4*B*). Second, when the roles of *locations X* and *Y* were exchanged during the experiment with high contrast alternating between them (Fig. 8), adaptation properties switched accordingly; for example, a locally adaptive cell showed faster and more biphasic filters when the corresponding local contrast was high, regardless of whether *locations X* or *Y* were considered (Figs. 8 and 10). However, future characterizations of the spatial scope of contrast adaptation for individual cells might be strengthened by additional recordings with varying placements of the stimulus subfields *X* and *Y*.

Apart from the example of transient OFF-alpha cells, we did not focus on a specific ganglion cell type, since our goal was an overall characterization of the spatial scope of contrast adaptation at the level of ganglion cells. Rather, our multielectrode-array recordings allowed us to assess a wide variety of ganglion cells, likely sampling many of the over 30 different types (Baden et al. 2016; Sanes and Masland 2015). This let us identify the multifaceted spatial scope of contrast adaptation and analyze its characteristics by selecting groups of cells from the edges of the continuous distribution of changes in temporal filtering. The results suggest that it may be useful to include the spatial scope of contrast adaptation in future detailed studies of specific, identified ganglion cell types.

#### Using local contrast changes to assess spatial adaptation features.

Our approach has been to apply visual stimuli with increased contrast in subregions of the receptive field (Garvert and Gollisch 2013). Similar approaches have also been used to distinguish adapting and sensitizing regions of ganglion cell receptive fields (Kastner and Baccus 2013) and to characterize spatially selective adaptation in the visual thalamus and in primary visual cortex (Dhruv and Carandini 2014). Analogously, the local increase in contrast may also be applied in an abstract stimulus space such as the orientation of visually displayed gratings (Benucci et al. 2013).

In our case, the stimuli and the contrast changes extended over the entire retina. This allowed us to simultaneously analyze cells spread across the entire spatial range of the multielectrode arrays. However, a disadvantage of this approach is that we cannot distinguish between effects occurring in the receptive field centers and effects occurring in the inhibitory surrounds. For example, it seems likely that the biphasic shape of the temporal filters is partly evoked by surround-triggered inhibition and that changes in biphasicness are thus related to contrast changes in the surround. Future studies may try differentiating between local contrast changes in the center and in the surround, perhaps in the context of single-cell recordings where receptive field locations are more easily accessible during the experiment. From a phenomenological point of view, however, the contrast changes in both center and surround may be considered a realistic scenario, as regions of higher and lower contrast in natural scenes will typically not be restricted to receptive field centers alone. Furthermore, our applied stimulus may be viewed as a straightforward extension of the frequently used approach of studying contrast adaptation through full-field stimulation (Shapley and Victor 1978; Smirnakis et al. 1997; Chander and Chichilnisky 2001; Kim and Rieke 2001; Baccus and Meister 2002), which thus naturally combines effects from center and surround.

#### LN model as a basis for analyzing contrast adaptation.

We based our analysis of the spatial scope of contrast adaptation on analyzing the ganglion cell activity in the framework of the linear-nonlinear (LN) model. This makes our results directly comparable to many previous investigations of contrast adaptation in the retina that apply this modeling framework (Baccus and Meister 2002; Beaudoin et al. 2007; Chander and Chichilnisky 2001; Garvert and Gollisch 2013; Kastner and Baccus 2011; Kerschensteiner et al. 2008; Kim and Rieke 2001; Liu and Gollisch 2015; Rieke 2001; Smirnakis et al. 1997; Zaghloul et al. 2005), though we here extended these analyses by adding spatial structure.

The LN model has proved to provide a fairly good representation of ganglion cell responses to white-noise stimulation in various species (Baccus and Meister 2002; Carandini et al. 2005; Chichilnisky 2001; Kim and Rieke 2001; Zaghloul et al. 2003), including mouse (Cui et al. 2016; Kerschensteiner et al. 2008; Nirenberg and Pandarinath 2012; Zhong et al. 2005), though improved models can be obtained by including feedback mechanisms or multiple filters with nonlinear interactions (Cui et al. 2016; Fairhall et al. 2006; Keat et al. 2001; Liu et al. 2017; McIntosh et al. 2016; Ozuysal and Baccus 2012; Pillow et al. 2005, 2008; Real et al. 2017; Schwartz et al. 2002). Often, the additional model components already partly capture phenomena of contrast adaptation, such as gain control or changes in temporal filtering. Thus, while the LN model does not capture some of the details of ganglion cell spike trains and is not the best available model for predicting ganglion cell responses, it commonly serves as framework for defining the characteristic features of signal processing, such as temporal filtering and sensitivity. This essentially establishes contrast-induced changes in parameters of the LN model as a useful “working definition” of contrast adaptation (Baccus and Meister 2002).

#### Connections to cellular and circuit mechanisms.

Our investigations aimed at a phenomenological description of how local contrast changes affect temporal filtering and sensitivity across the receptive field of individual ganglion cells. Yet, differences in the spatial scope are likely linked to differences in mechanistic components of contrast adaptation and may thus help distinguish which mechanisms predominate in which ganglion cells.

Contrast adaptation in the retina appears to primarily result from synaptic depression at the bipolar-to-ganglion cell synapse (Burrone and Lagnado 2000; Jarsky et al. 2011; Li et al. 2007; Nikolaev et al. 2013; Singer and Diamond 2006) and from intrinsic mechanisms of the ganglion cell, such as inactivation of sodium currents (Kim and Rieke 2003) and activation of potassium currents (Weick and Demb 2011). Such cell-intrinsic mechanisms should lead to global adaptation and are most strongly triggered by the cell’s own spiking activity. This fits well with our observation that globally adaptive cells displayed the strongest relative increase in firing rate when local contrast increased (Fig. 3*H*), suggesting that spiking-related, ganglion cell-intrinsic adaptation mechanisms are particularly strongly triggered in these cells. Conversely, the small differences in steady-state firing observed for fixed-filter cells is likely one reason why these cells generally show little contrast adaptation. The small effect of contrast on the average firing rate in these cells is likely a reflection of a particular linear relation between contrast and evoked firing rate.

Synaptic depression between bipolar and ganglion cells, on the other hand, is expected to contribute to local adaptation and may thus be a primary mechanism in locally adaptive cells. Local increases in contrast would here lead to increased activity in a subset of presynaptic bipolar cells, which have smaller receptive fields than ganglion cells. The ensuing synaptic depression at the output synapses of these bipolar cells then results in a local reduction in gain (Ozuysal and Baccus 2012). Alternatively, local adaptation might by inherited from adaptation in the membrane potential of bipolar cells (Rieke 2001), though the extent to which bipolar cell membrane potentials may show contrast adaptation is still unclear and could depend on the subtype of bipolar cell (Baccus and Meister 2002). Note, however, that local mechanisms, such as synaptic depression may also lead to functionally global adaptation effects (Garvert and Gollisch 2013). As basal transmitter release decreases (Beaudoin et al. 2007; Manookin and Demb 2006), the postsynaptic ganglion cell may become relatively hyperpolarized (Baccus and Meister 2002; Manookin and Demb 2006), which effectively increases the threshold for stimulation at any location in the receptive field, corresponding to a global sensitivity change.

A peculiar finding of the present study was the observation of translocally adaptive cells, which have stronger filter changes for locations where contrast did not change. These filter changes manifested themselves primarily in the increased biphasic filter shape when contrast was high at other locations. We have previously shown that such perhaps unexpected contrast dependence of biphasicness may result when local nonlinearities of signal transmission precede a negative feedback process (Garvert and Gollisch 2013). In essence, the negative feedback process triggered by stimulation at one location is more effective when contrast is high at other locations because the increased activity that is triggered by these other locations provides for more opportunities to suppress activity. This then leads to a more pronounced secondary filter peak when contrast is high at other locations.

#### Receptive field substructure.

The spatial characteristics of retinal contrast adaptation appear to be strongly related to the nonlinearities of stimulus processing. Global adaptation properties, for example, were found to be strongly related to large changes in average firing rates between different contrast conditions (Fig. 3*H*), and these firing rate differences were likely a consequence of how linear or nonlinear the cells were in their responses to luminance changes (Fig. 3*I*). Furthermore, local nonlinearities in the signal transmission between bipolar and ganglion cell are important for turning synaptic dynamics into actual variance-adaptation mechanisms (Jarsky et al. 2011; Ozuysal and Baccus 2012) and shape how ganglion cells respond to sudden switches in local contrast (Garvert and Gollisch 2013).

Thus, the question of spatial scope of contrast adaptation ties in closely with nonlinear signal pooling across the receptive field. For many ganglion cells, receptive field centers are structured into subunits, corresponding to individual presynaptic bipolar cells (Demb et al. 2001; Liu et al. 2017; Schwartz et al. 2012). Nonlinear signal pooling over these subunits is thought to be essential for the computations occurring in the retinal circuit (Bölinger and Gollisch 2012; Gollisch and Meister 2010; Roska and Meister 2014) and for the encoding of complex, natural stimuli (Gollisch 2013; Schwartz and Rieke 2011; Schwartz et al. 2012). In this context, the question of local vs. global contrast adaptation amounts to asking whether subunits can adapt independently or always do so in unison. Understanding this will help us connect the mechanisms of adaptation to the specific, nonlinear functions of different ganglion cell types for the encoding of spatially structured, dynamic visual stimuli.

## GRANTS

This work was supported by the Deutsche Forschungsgemeinschaft through the Collaborative Research Center 889 “Cellular Mechanisms of Sensory Processing,” project C1, and by the European Research Council (ERC) under the European Union’s Horizon 2020 research and innovation program (grant agreement number 724822).

## DISCLOSURES

No conflicts of interest, financial or otherwise, are declared by the authors.

## AUTHOR CONTRIBUTIONS

M.H.K. and T.G. conceived and designed research; M.H.K. performed experiments; M.H.K. analyzed data; M.H.K. and T.G. interpreted results of experiments; M.H.K. prepared figures; M.H.K. and T.G. drafted manuscript; M.H.K. and T.G. edited and revised manuscript; M.H.K. and T.G. approved final version of manuscript.

## References

[B1] Baccus SA, Meister M Fast and slow contrast adaptation in retinal circuitry. Neuron 36: 909–919, 2002. doi:10.1016/S0896-6273(02)01050-4. 12467594

[B2] Baden T, Berens P, Franke K, Román Rosón M, Bethge M, Euler T The functional diversity of retinal ganglion cells in the mouse. Nature 529: 345–350, 2016. doi:10.1038/nature16468. 26735013 PMC4724341

[B3] Beaudoin DL, Borghuis BG, Demb JB Cellular basis for contrast gain control over the receptive field center of mammalian retinal ganglion cells. J Neurosci 27: 2636–2645, 2007. doi:10.1523/JNEUROSCI.4610-06.2007. 17344401 PMC6672510

[B4] Benucci A, Saleem AB, Carandini M Adaptation maintains population homeostasis in primary visual cortex. Nat Neurosci 16: 724–729, 2013. doi:10.1038/nn.3382. 23603708 PMC3665725

[B5] Berntson A, Taylor WR Response characteristics and receptive field widths of on-bipolar cells in the mouse retina. J Physiol 524: 879–889, 2000. doi:10.1111/j.1469-7793.2000.00879.x. 10790165 PMC2269911

[B6] Berry MJ II, Brivanlou IH, Jordan TA, Meister M Anticipation of moving stimuli by the retina. Nature 398: 334–338, 1999. doi:10.1038/18678. 10192333

[B7] Berry MJ II, Meister M Refractoriness and neural precision. J Neurosci 18: 2200–2211, 1998. 9482804 10.1523/JNEUROSCI.18-06-02200.1998 PMC6792934

[B8] Bölinger D, Gollisch T Closed-loop measurements of iso-response stimuli reveal dynamic nonlinear stimulus integration in the retina. Neuron 73: 333–346, 2012. doi:10.1016/j.neuron.2011.10.039. 22284187

[B9] Brown SP, Masland RH Spatial scale and cellular substrate of contrast adaptation by retinal ganglion cells. Nat Neurosci 4: 44–51, 2001. doi:10.1038/82888. 11135644

[B10] Burrone J, Lagnado L Synaptic depression and the kinetics of exocytosis in retinal bipolar cells. J Neurosci 20: 568–578, 2000. 10632586 10.1523/JNEUROSCI.20-02-00568.2000 PMC6772421

[B11] Carandini M, Demb JB, Mante V, Tolhurst DJ, Dan Y, Olshausen BA, Gallant JL, Rust NC Do we know what the early visual system does? J Neurosci 25: 10577–10597, 2005. doi:10.1523/JNEUROSCI.3726-05.2005. 16291931 PMC6725861

[B12] Carandini M, Ferster D A tonic hyperpolarization underlying contrast adaptation in cat visual cortex. Science 276: 949–952, 1997. doi:10.1126/science.276.5314.949. 9139658

[B13] Chander D, Chichilnisky EJ Adaptation to temporal contrast in primate and salamander retina. J Neurosci 21: 9904–9916, 2001. 11739598 10.1523/JNEUROSCI.21-24-09904.2001 PMC6763043

[B14] Chen EY, Chou J, Park J, Schwartz G, Berry MJ II The neural circuit mechanisms underlying the retinal response to motion reversal. J Neurosci 34: 15557–15575, 2014. doi:10.1523/JNEUROSCI.1460-13.2014. 25411485 PMC4236393

[B15] Chichilnisky EJ A simple white noise analysis of neuronal light responses. Network 12: 199–213, 2001. doi:10.1080/713663221. 11405422

[B16] Cui Y, Wang YV, Park SJ, Demb JB, Butts DA Divisive suppression explains high-precision firing and contrast adaptation in retinal ganglion cells. eLife 5: e19460, 2016. doi:10.7554/eLife.19460. 27841746 PMC5108594

[B17] Dean I, Harper NS, McAlpine D Neural population coding of sound level adapts to stimulus statistics. Nat Neurosci 8: 1684–1689, 2005. doi:10.1038/nn1541. 16286934

[B18] Demb JB Multiple mechanisms for contrast adaptation in the retina. Neuron 36: 781–783, 2002. doi:10.1016/S0896-6273(02)01100-5. 12467580

[B19] Demb JB Functional circuitry of visual adaptation in the retina. J Physiol 586: 4377–4384, 2008. doi:10.1113/jphysiol.2008.156638. 18617564 PMC2614018

[B20] Demb JB, Zaghloul K, Haarsma L, Sterling P Bipolar cells contribute to nonlinear spatial summation in the brisk-transient (Y) ganglion cell in mammalian retina. J Neurosci 21: 7447–7454, 2001. 11567034 10.1523/JNEUROSCI.21-19-07447.2001 PMC6762908

[B21] Dhruv NT, Carandini M Cascaded effects of spatial adaptation in the early visual system. Neuron 81: 529–535, 2014. doi:10.1016/j.neuron.2013.11.025. 24507190 PMC3969249

[B22] Fairhall AL, Burlingame CA, Narasimhan R, Harris RA, Puchalla JL, Berry MJ II Selectivity for multiple stimulus features in retinal ganglion cells. J Neurophysiol 96: 2724–2738, 2006. doi:10.1152/jn.00995.2005. 16914609

[B23] Garcia-Lazaro JA, Ho SS, Nair A, Schnupp JW Shifting and scaling adaptation to dynamic stimuli in somatosensory cortex. Eur J Neurosci 26: 2359–2368, 2007. doi:10.1111/j.1460-9568.2007.05847.x. 17953623

[B24] Garvert MM, Gollisch T Local and global contrast adaptation in retinal ganglion cells. Neuron 77: 915–928, 2013. doi:10.1016/j.neuron.2012.12.030. 23473321

[B25] Gauthier JL, Field GD, Sher A, Greschner M, Shlens J, Litke AM, Chichilnisky EJ Receptive fields in primate retina are coordinated to sample visual space more uniformly. PLoS Biol 7: e1000063, 2009. doi:10.1371/journal.pbio.1000063. 19355787 PMC2672597

[B26] Gollisch T Features and functions of nonlinear spatial integration by retinal ganglion cells. J Physiol Paris 107: 338–348, 2013. doi:10.1016/j.jphysparis.2012.12.001. 23262113

[B27] Gollisch T, Meister M Eye smarter than scientists believed: neural computations in circuits of the retina. Neuron 65: 150–164, 2010. doi:10.1016/j.neuron.2009.12.009. 20152123 PMC3717333

[B28] Jarsky T, Cembrowski M, Logan SM, Kath WL, Riecke H, Demb JB, Singer JH A synaptic mechanism for retinal adaptation to luminance and contrast. J Neurosci 31: 11003–11015, 2011. doi:10.1523/JNEUROSCI.2631-11.2011. 21795549 PMC3152984

[B29] Kastner DB, Baccus SA Coordinated dynamic encoding in the retina using opposing forms of plasticity. Nat Neurosci 14: 1317–1322, 2011. doi:10.1038/nn.2906. 21909086 PMC3359137

[B30] Kastner DB, Baccus SA Spatial segregation of adaptation and predictive sensitization in retinal ganglion cells. Neuron 79: 541–554, 2013. doi:10.1016/j.neuron.2013.06.011. 23932000 PMC4046856

[B31] Keat J, Reinagel P, Reid RC, Meister M Predicting every spike: a model for the responses of visual neurons. Neuron 30: 803–817, 2001. doi:10.1016/S0896-6273(01)00322-1. 11430813

[B32] Kerschensteiner D, Liu H, Cheng CW, Demas J, Cheng SH, Hui CC, Chow RL, Wong RO Genetic control of circuit function: Vsx1 and Irx5 transcription factors regulate contrast adaptation in the mouse retina. J Neurosci 28: 2342–2352, 2008. doi:10.1523/JNEUROSCI.4784-07.2008. 18322081 PMC6671180

[B33] Kim KJ, Rieke F Temporal contrast adaptation in the input and output signals of salamander retinal ganglion cells. J Neurosci 21: 287–299, 2001. 11150346 10.1523/JNEUROSCI.21-01-00287.2001 PMC6762442

[B34] Kim KJ, Rieke F Slow Na+ inactivation and variance adaptation in salamander retinal ganglion cells. J Neurosci 23: 1506–1516, 2003. 12598639 10.1523/JNEUROSCI.23-04-01506.2003 PMC6742238

[B35] Koehler CL, Akimov NP, Rentería RC Receptive field center size decreases and firing properties mature in ON and OFF retinal ganglion cells after eye opening in the mouse. J Neurophysiol 106: 895–904, 2011. doi:10.1152/jn.01046.2010. 21613583 PMC3154829

[B36] Korenberg MJ, Hunter IW The identification of nonlinear biological systems: LNL cascade models. Biol Cybern 55: 125–134, 1986. 3801533 10.1007/BF00341928

[B37] Krishnamoorthy V, Weick M, Gollisch T Sensitivity to image recurrence across eye-movement-like image transitions through local serial inhibition in the retina. eLife 6: e22431, 2017. doi:10.7554/eLife.22431. 28230526 PMC5338922

[B38] Kvale MN, Schreiner CE Short-term adaptation of auditory receptive fields to dynamic stimuli. J Neurophysiol 91: 604–612, 2004. doi:10.1152/jn.00484.2003. 14762146

[B39] Leonardo A, Meister M Nonlinear dynamics support a linear population code in a retinal target-tracking circuit. J Neurosci 33: 16971–16982, 2013. doi:10.1523/JNEUROSCI.2257-13.2013. 24155302 PMC3807026

[B40] Levy M, Fournier J, Frégnac Y The role of delayed suppression in slow and fast contrast adaptation in V1 simple cells. J Neurosci 33: 6388–6400, 2013. doi:10.1523/JNEUROSCI.3609-12.2013. 23575837 PMC6619067

[B41] Li GL, Vigh J, von Gersdorff H Short-term depression at the reciprocal synapses between a retinal bipolar cell terminal and amacrine cells. J Neurosci 27: 7377–7385, 2007. doi:10.1523/JNEUROSCI.0410-07.2007. 17626198 PMC6672600

[B42] Liu JK, Gollisch T Spike-triggered covariance analysis reveals phenomenological diversity of contrast adaptation in the retina. PLOS Comput Biol 11: e1004425, 2015. doi:10.1371/journal.pcbi.1004425. 26230927 PMC4521887

[B43] Liu JK, Schreyer HM, Onken A, Rozenblit F, Khani MH, Krishnamoorthy V, Panzeri S, Gollisch T Inference of neuronal functional circuitry with spike-triggered non-negative matrix factorization. Nat Commun 8: 149, 2017. doi:10.1038/s41467-017-00156-9. 28747662 PMC5529558

[B44] Maffei L, Fiorentini A, Bisti S Neural correlate of perceptual adaptation to gratings. Science 182: 1036–1038, 1973. doi:10.1126/science.182.4116.1036. 4748674

[B45] Manookin MB, Demb JB Presynaptic mechanism for slow contrast adaptation in mammalian retinal ganglion cells. Neuron 50: 453–464, 2006. doi:10.1016/j.neuron.2006.03.039. 16675399

[B46] Maravall M, Petersen RS, Fairhall AL, Arabzadeh E, Diamond ME Shifts in coding properties and maintenance of information transmission during adaptation in barrel cortex. PLoS Biol 5: e19, 2007. doi:10.1371/journal.pbio.0050019. 17253902 PMC1779810

[B47] McIntosh LT, Maheswaranathan N, Nayebi A, Ganguli S, Baccus SA Deep learning models of the retinal response to natural scenes. Adv Neural Inf Process Syst 29: 1369–1377, 2016. 28729779 PMC5515384

[B48] Movshon JA, Lennie P Pattern-selective adaptation in visual cortical neurones. Nature 278: 850–852, 1979. doi:10.1038/278850a0. 440411

[B49] Nagel KI, Doupe AJ Temporal processing and adaptation in the songbird auditory forebrain. Neuron 51: 845–859, 2006. doi:10.1016/j.neuron.2006.08.030. 16982428

[B50] Nikolaev A, Leung KM, Odermatt B, Lagnado L Synaptic mechanisms of adaptation and sensitization in the retina. Nat Neurosci 16: 934–941, 2013. doi:10.1038/nn.3408. 23685718 PMC3924174

[B51] Nirenberg S, Pandarinath C Retinal prosthetic strategy with the capacity to restore normal vision. Proc Natl Acad Sci USA 109: 15012–15017, 2012. doi:10.1073/pnas.1207035109. 22891310 PMC3443127

[B52] Ozuysal Y, Baccus SA Linking the computational structure of variance adaptation to biophysical mechanisms. Neuron 73: 1002–1015, 2012. doi:10.1016/j.neuron.2011.12.029. 22405209 PMC4046855

[B53] Paninski L Convergence properties of three spike-triggered analysis techniques. Network 14: 437–464, 2003. doi:10.1088/0954-898X_14_3_304. 12938766

[B54] Pillow JW, Paninski L, Uzzell VJ, Simoncelli EP, Chichilnisky EJ Prediction and decoding of retinal ganglion cell responses with a probabilistic spiking model. J Neurosci 25: 11003–11013, 2005. doi:10.1523/JNEUROSCI.3305-05.2005. 16306413 PMC6725882

[B55] Pillow JW, Shlens J, Paninski L, Sher A, Litke AM, Chichilnisky EJ, Simoncelli EP Spatio-temporal correlations and visual signalling in a complete neuronal population. Nature 454: 995–999, 2008. doi:10.1038/nature07140. 18650810 PMC2684455

[B56] Pouzat C, Mazor O, Laurent G Using noise signature to optimize spike-sorting and to assess neuronal classification quality. J Neurosci Methods 122: 43–57, 2002. doi:10.1016/S0165-0270(02)00276-5. 12535763

[B57] Real E, Asari H, Gollisch T, Meister M Neural circuit inference from function to structure. Curr Biol 27: 189–198, 2017. doi:10.1016/j.cub.2016.11.040. 28065610 PMC5821114

[B58] Rieke F Temporal contrast adaptation in salamander bipolar cells. J Neurosci 21: 9445–9454, 2001. 11717378 10.1523/JNEUROSCI.21-23-09445.2001 PMC6763932

[B59] Rieke F, Rudd ME The challenges natural images pose for visual adaptation. Neuron 64: 605–616, 2009. doi:10.1016/j.neuron.2009.11.028. 20005818

[B60] Roska B, Meister M The retina dissects the visual scene into distinct features. In: The New Visual Neurosciences, edited by Werner JS, Chalupa LM Cambridge, MA: MIT Press, 2014, p. 163–182.

[B61] Sakai HM, Naka K, Korenberg MJ White-noise analysis in visual neuroscience. Vis Neurosci 1: 287–296, 1988. doi:10.1017/S0952523800001942. 3154801

[B62] Samengo I, Gollisch T Spike-triggered covariance: geometric proof, symmetry properties, and extension beyond Gaussian stimuli. J Comput Neurosci 34: 137–161, 2013. doi:10.1007/s10827-012-0411-y. 22798148 PMC3558678

[B63] Sanes JR, Masland RH The types of retinal ganglion cells: current status and implications for neuronal classification. Annu Rev Neurosci 38: 221–246, 2015. doi:10.1146/annurev-neuro-071714-034120. 25897874

[B64] Schwartz G, Rieke F Nonlinear spatial encoding by retinal ganglion cells: when 1 + 1 ≠ 2. J Gen Physiol 138: 283–290, 2011. doi:10.1085/jgp.201110629. 21875977 PMC3171084

[B65] Schwartz GW, Okawa H, Dunn FA, Morgan JL, Kerschensteiner D, Wong RO, Rieke F The spatial structure of a nonlinear receptive field. Nat Neurosci 15: 1572–1580, 2012. doi:10.1038/nn.3225. 23001060 PMC3517818

[B66] Schwartz O, Chichilnisky EJ, Simoncelli EP Characterizing neural gain control using spike-triggered covariance. Adv Neural Inf Process Syst 14: 269–276, 2002.

[B67] Schwartz O, Pillow JW, Rust NC, Simoncelli EP Spike-triggered neural characterization. J Vis 6: 484–507, 2006. doi:10.1167/6.4.13. 16889482

[B68] Shapley RM, Victor JD The effect of contrast on the transfer properties of cat retinal ganglion cells. J Physiol 285: 275–298, 1978. doi:10.1113/jphysiol.1978.sp012571. 745079 PMC1281756

[B69] Shapley RM, Victor JD Nonlinear spatial summation and the contrast gain control of cat retinal ganglion cells. J Physiol 290: 141–161, 1979. doi:10.1113/jphysiol.1979.sp012765. 469742 PMC1278829

[B70] Shapley RM, Victor JD How the contrast gain control modifies the frequency responses of cat retinal ganglion cells. J Physiol 318: 161–179, 1981. doi:10.1113/jphysiol.1981.sp013856. 7320887 PMC1245483

[B71] Singer JH, Diamond JS Vesicle depletion and synaptic depression at a mammalian ribbon synapse. J Neurophysiol 95: 3191–3198, 2006. doi:10.1152/jn.01309.2005. 16452253

[B72] Smirnakis SM, Berry MJ, Warland DK, Bialek W, Meister M Adaptation of retinal processing to image contrast and spatial scale. Nature 386: 69–73, 1997. doi:10.1038/386069a0. 9052781

[B73] Solomon SG, Peirce JW, Dhruv NT, Lennie P Profound contrast adaptation early in the visual pathway. Neuron 42: 155–162, 2004. doi:10.1016/S0896-6273(04)00178-3. 15066272

[B74] Wark B, Fairhall A, Rieke F Timescales of inference in visual adaptation. Neuron 61: 750–761, 2009. doi:10.1016/j.neuron.2009.01.019. 19285471 PMC2677143

[B75] Weick M, Demb JB Delayed-rectifier K channels contribute to contrast adaptation in mammalian retinal ganglion cells. Neuron 71: 166–179, 2011. doi:10.1016/j.neuron.2011.04.033. 21745646 PMC3134798

[B76] Wolfe J, Palmer LA Temporal diversity in the lateral geniculate nucleus of cat. Vis Neurosci 15: 653–675, 1998. doi:10.1017/S0952523898154068. 9682868

[B77] Zaghloul KA, Boahen K, Demb JB Different circuits for ON and OFF retinal ganglion cells cause different contrast sensitivities. J Neurosci 23: 2645–2654, 2003. 12684450 10.1523/JNEUROSCI.23-07-02645.2003 PMC6742092

[B78] Zaghloul KA, Boahen K, Demb JB Contrast adaptation in subthreshold and spiking responses of mammalian Y-type retinal ganglion cells. J Neurosci 25: 860–868, 2005. doi:10.1523/JNEUROSCI.2782-04.2005. 15673666 PMC6725633

[B79] Zaghloul KA, Manookin MB, Borghuis BG, Boahen K, Demb JB Functional circuitry for peripheral suppression in mammalian Y-type retinal ganglion cells. J Neurophysiol 97: 4327–4340, 2007. doi:10.1152/jn.01091.2006. 17460102

[B80] Zhong Q, Roychowdhury V, Boykin P, Jacobs A, Nirenberg S A filter based encoding model for mouse retinal ganglion cells. Conf Proc IEEE Eng Med Biol Soc 2: 2087–2090, 2005. doi:10.1109/IEMBS.2005.1616870. 17282639

